# An umbrella review and meta‐analysis of renin–angiotensin system drugs use and COVID‐19 outcomes

**DOI:** 10.1111/eci.13888

**Published:** 2022-10-19

**Authors:** Amanj Kurdi, Tanja Mueller, Natalie Weir

**Affiliations:** ^1^ Strathclyde Institute of Pharmacy and Biomedical Science, University of Strathclyde Glasgow Scotland UK; ^2^ Department of Pharmacology and Toxicology College of Pharmacy, Kurdistan Region Government, Hawler Medical University Erbil Iraq; ^3^ Division of Public Health Pharmacy and Management School of Pharmacy, Sefako Makgatho Health Sciences University Pretoria South Africa

**Keywords:** angiotensin receptor II blockers (ARBs), angiotensin‐converting enzyme inhibitors (ACEIs), COVID‐19, renin–angiotensin–aldosterone system (RAAS) inhibitors, umbrella review

## Abstract

**Background:**

Despite the availability of extensive literature on the effect of angiotensin‐converting enzyme inhibitors (ACEIs)/angiotensin‐receptor blockers (ARBs) on COVID‐19 outcomes, the evidence is still controversial. We aimed to provide a comprehensive assessment of the effect of ACEIs/ARBs on COVID‐19‐related outcomes by summarising the currently available evidence.

**Methods:**

An umbrella review was conducted using Medline (OVID), Embase, Scopus, Cochrane library and medRxiv from inception to 1 February 2021. Systematic reviews with meta‐analysis that evaluated the effect of ACEIs/ARBs on COVID‐19‐related clinical outcomes were eligible. Studies' quality was appraised using the AMSTAR 2 Critical Appraisal Tool. Data were analysed using the random‐effects modelling including several subgroup analyses. Heterogenicity was assessed using I^2^ statistic. The study protocol was registered in PROSPERO (CRD42021233398) and reported using PRISMA guidelines.

**Results:**

Overall, 47 reviews were eligible for inclusion. Out of the nine COVID‐19 outcomes evaluated, there was significant associations between ACEIs/ARBs use and each of death (OR = 0.80, 95%CI = 0.75–0.86; I^2^ = 51.9%), death/ICU admission as composite outcome (OR = 0.86, 95%CI = 0.80–0.92; I^2^ = 43.9%), severe COVID‐19 (OR = 0.86, 95%CI = 0.78–0.95; I^2^ = 68%) and hospitalisation (OR = 1.23, 95%CI = 1.04–1.46; I^2^ = 76.4%). The significant reduction in death/ICU admission, however, was higher among studies which presented adjusted measure of effects (OR = 0.63, 95%CI = 0.47–0.84) and were of moderate quality (OR = 0.74, 95%CI = 0.63–0.85).

**Conclusions:**

Collective evidence from observational studies indicate a good quality evidence on the significant association between ACEIs/ARBs use and reduction in death and death/ICU admission, but poor‐quality evidence on both reducing severe COVID‐19 and increasing hospitalisation. Our findings further support the current recommendations of not discontinuing ACEIs/ARBs therapy in patients with COVID‐19.

## 1 INTRODUCTION

Several risk factors linked to poor COVID‐19 outcomes have been identified early on, including cardiovascular diseases such as hypertension.^1^ Consequently, the possible impact of renin–angiotensin–aldosterone system (RAAS) inhibitors on COVID‐19‐related outcomes has emerged as a topic of interest^2^ and their mechanisms of action– in particular, the potential upregulation of angiotensin‐converting enzyme 2 (ACE2), which is associated with viral entry into bronchial cells.^3^ This has resulted in the rapid dissemination of numerous studies, mostly retrospective observational in nature, focussing on the risk of COVID‐19 infection, disease severity and/or disease outcomes in patients being treated with either angiotensin‐converting‐enzyme inhibitors (ACEIs)/angiotensin receptor blockers (ARBs) since early 2020.^4^, ^5^, ^6^

As was the case in most early COVID‐19‐related research, the evidence comprised observational studies with notably small sample sizes and short durations of follow‐up. Resultantly, a number of systematic reviews were swiftly published in an attempt to offer a more substantial view by aggregating findings of these small‐scale studies. These meta‐analyses have offered tentative insights into all three areas of interest with regard to the use of RAAS inhibitors in times of COVID‐19: (i) risk of infection, usually measured as the share of positive PCR tests within a study cohort; (ii) risk of severe COVID‐19, with various underlying definitions ranging from hospitalisation due to the disease to the requirement for mechanical ventilation; and (iii) the risk of mortality. While there were similarities between some of the published results—for example, indicating, in general, no association between RAAS inhibitor use and risk of COVID‐19 infection—other results were more varied and the findings are still controversial/conflicting.^4^, ^5^, ^6^ A logical next step, besides conducting additional systematic reviews/meta‐analyses, is to perform a systematic review of systematic reviews (also known as umbrella review), thereby taking advantage of the availability of high‐level evidence and providing an opportunity to contrast and compare.^7^ The aim of this umbrella review and meta‐analysis, therefore, was to assess the effect of ACEIs/ARBs on COVID‐19‐related outcomes by summarising the currently available, aggregate evidence.

## 2 METHODS

An umbrella literature review and subsequent meta‐analysis was conducted. The protocol was informed by Joanna Briggs Reviewer's Manual for ‘Development of an Umbrella review protocol’^8^ and published on PROSPERO (CRD42021233398).

### 2.1 Eligibility criteria

Eligible studies were systematic reviews, which conducted a meta‐analysis to explore the effect of ACEIs/ARBs on any COVID‐19‐related clinical outcomes among adults (≥18 years) with COVID‐19 diagnosis.

### 2.2 Search strategy

The databases Medline, EMBASE, Scopus, Cochrane and medRxiv were searched from January 2019 until February 2021. Furthermore, we have performed a further scoping updated search in September 2022 to identify any potentially eligible studies published after our original search date. The search was limited to the English language and for systematic review articles. Search terms are listed in Supplementary file S1.

### 2.3 Article selection

Article selection was conducted using Covidence software^9^; 10% of the articles' titles/abstracts and full texts were randomly selected and screened independently. The percentage of agreement was calculated for all independent validation, with >80% considered adequate.^10^

### 2.4 Data extraction

A data extraction template in Microsoft Excel was piloted with 10% of reviews by NW and agreed for use by all authors. 10% of reviews were randomly selected and underwent independent data extraction; the percentage of agreement was calculated. Again, agreement >80% was considered adequate^10^. Data extracted from the reviews included title; authors; year review published; study design; sample size; setting; population; exposure (e.g. ACEIs/ARBs, ACEIs or ARBs) and outcomes (e.g. death, COVID‐19 infection and hospitalisation).

### 2.5 Quality assessment

Quality assessment was conducted independently using the AMSTAR 2 tool.^11^ Studies were assessed based on the 15 AMSTAR 2 domains. To determine the overall confidence in the results of the review, studies were categorised as having high, moderate, low and critically low confidence in the results. As per AMSTAR 2 guidance, the overall confidence in the results was calculated based on the number of critical and noncritical domains. For this review, there were four critical domains: if there was an explicit statement that the methods were established a priori within a protocol; if a satisfactory technique for assessing the risk of bias was conducted and sufficiently discussed; if the meta‐analysis used appropriate methods; and if publication bias (small study bias) was conducted. If the criteria of a critical domain are not met, then this indicates a critical weakness in the review. The remaining 11 domains were considered noncritical. As per AMSTAR 2 guidance, reviews were classified as having high overall confidence in the results if there was ≤1 noncritical weakness, moderate if there was >1 noncritical weakness, low if there was 1 critical weakness and critically low if there >1 critical weakness.

### 2.6 Data analysis and synthesis

The random‐effects meta‐analysis model was used to statistically combine the measure of effects for those outcomes that were reported by more than one study, stratified by the three levels of exposure (ACEIs/ARBs, ACEIs and ARBs). In order to explore potential sources of heterogeneity, we conducted several subgroup analyses based on numerous variables including whether the reported measure of effects was crude or adjusted, the study was peer‐reviewed or not, and the study's methodological quality as per the quality assessment. Furthermore, to assess the impact of ACEIs/ARBs among patients with hypertension (the most common indication for ACEIs/ARBs), we also conducted subgroup analysis based on whether the studies had included either patients with hypertension only or at least had hypertension as one of the comorbidities versus those studies which did not record the hypertension status of their study population. In order to account for any possibility of rising in type I error (resulted from the multiple subgroup meta‐analyses), we adopted a lower significance threshold of <0.02 (2%) instead of <0.05 (5%) as a sensitivity analysis. The combined pooled estimates were presented as odds ratios and 95%CI and graphically as forest plots. I^2^ statistic^12^ was used to assess heterogeneity between the studies with I^2^ of 0% indicating a lack of heterogeneity, whereas 25%, 50% and 75% indicating low, moderate and high heterogeneity, respectively.^12^ To evaluate the degree of overlap of the studies within the included reviews, a citation matrix was generated that was used to calculate the Corrected Cover Area (CCA) as suggested by Pieper et al.^13^ Publication bias was assessed using funnel plots and Egger's asymmetry test^14^ only for those outcomes where >10 studies were included in the analysis as recommended by Cochrane guidelines.^15^ Furthermore, we evaluated the influence of individual reviews on the summary pooled estimate for each outcome by conducting influential analyses^16^ whereby the pooled meta‐analysis estimates for each outcome were computed by omitting one study at a time. Data were analysed using STATA 12.

### 2.7 Role of the funding source

None.

## 3 RESULTS

**FIGURE 1:**
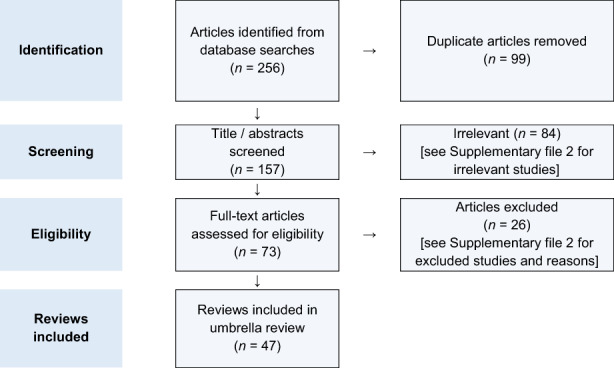
PRISMA flow diagram of the review selection process

Out of an initial 157 publications, 66 systematic reviews underwent full‐text screening; after further exclusions based on prespecified criteria, 47 studies were eligible for inclusion (Figure 1).^4^, ^5^, ^6^, ^17^, ^18^, ^19^, ^20^, ^21^, ^22^, ^23^, ^24^, ^25^, ^26^, ^27^, ^28^, ^29^, ^30^, ^31^, ^32^, ^33^, ^34^, ^35^, ^36^, ^37^, ^38^, ^39^, ^40^, ^41^, ^42^, ^43^, ^44^, ^45^, ^46^, ^47^, ^48^, ^49^, ^50^, ^51^, ^52^, ^53^, ^54^, ^55^, ^56^, ^57^, ^58^, ^59^, ^60^ Through the further scoping search in September 2022, we could identify three additional studies^61^, ^62^, ^63^ of relevance, published after our original search date of 1 February 2021, yet these were not eligible for inclusion due to several reasons. First, the study by Iheanacho CO et al^61^ was a systemic review without a meta‐analysis, which makes it ineligible for inclusion in our umbrella review. Second, the other two studies by Laurentius A et al^62^ and Singh R et al^63^ had a high likelihood of overlap and duplication with the reviews already included in our umbrella review because their search end dates were either earlier or very close to the search end dates of some other reviews included in our umbrella review. Third, the findings of these identified studies offer comparable conclusions to our umbrella review and would have only contributed to the pooled estimates of two out of the nine outcomes analysed in our umbrella review. Therefore, these studies were not considered because we believe it would offer little, if any, further insight into the current landscape of evidence and will have no substantial impact on the conclusions drawn.

### 3.1 Review characteristics

Forty‐six reviews (97.9%) compared COVID‐19‐related outcomes between ACEI/ARB users vs. nonusers among patients with COVID‐19,^4^, ^5^, ^6^, ^17^, ^18^, ^19^, ^20^, ^21^, ^22^, ^23^, ^24^, ^25^, ^26^, ^27^, ^28^, ^29^, ^30^, ^31^, ^32^, ^33^, ^34^, ^35^, ^36^, ^37^, ^38^, ^39^, ^40^, ^41^, ^42^, ^43^, ^44^, ^45^, ^46^, ^47^, ^48^, ^49^, ^50^, ^51^, ^52^, ^54^, ^55^, ^56^, ^57^, ^58^, ^59^, ^60^ one study (2.12%) compared outcomes between ACEIs/ARBs users in patients with and without COVID‐19 infection^53^, and 16 studies (34.0%) explored both.^6^, ^19^, ^25^, ^26^, ^27^, ^40^, ^41^, ^43^, ^44^, ^48^, ^50^, ^51^, ^54^, ^56^, ^58^, ^60^ Definition criteria for COVID‐19 diagnosis were reported by only six (12.8%) reviews as laboratory‐confirmed diagnosis based on a reverse transcriptase–polymerase chain reaction, whereas the remaining 41 (87.2%) reviews did not report any criteria for COVID‐19 diagnosis definition. Most of the included reviews were peer‐reviewed publications (68.1%; *n* = 32), whereas the remaining 15 (31.9%) reviews were non‐peer‐reviewed publications (i.e. were published in a preprint database).^17^, ^18^, ^19^, ^21^, ^22^, ^23^, ^30^, ^32^, ^33^, ^34^, ^36^, ^46^, ^50^, ^54^, ^60^ The time the searches were conducted ranged from April 2020 to October 2020, with 21 (44.7%) review searches conducted in the month of May 2020^4^, ^5^, ^6^, ^17^, ^21^, ^23^, ^24^, ^28^, ^30^, ^31^, ^32^, ^35^, ^36^, ^40^, ^41^, ^42^, ^44^, ^46^, ^48^, ^50^, ^54^ Preprint articles were included in 28 (59.6%) reviews,^4^, ^17^, ^19^, ^20^, ^21^, ^22^, ^25^, ^26^, ^30^, ^33^, ^37^, ^41^, ^42^, ^43^, ^44^, ^45^, ^47^, ^48^, ^49^, ^50^, ^51^, ^52^, ^53^, ^55^, ^56^, ^59^, ^60^ and 10 (21.3%) reviews adjusted for retracted studies.^4^, ^18^, ^31^, ^40^, ^45^, ^47^, ^48^, ^49^, ^50^, ^56^ Full details of the 47 reviews are presented in Supplementary file S3.

A total of 213 meta‐analyses were conducted by the 47 reviews (Supplementary file S4). In terms of number of COVID‐19‐related outcomes reported in each review, one outcome was reported by 13 reviews (27.7%),^5^, ^18^, ^20^, ^21^, ^23^, ^24^, ^28^, ^29^, ^38^, ^39^, ^47^, ^52^, ^53^ two outcomes by 15 reviews (31.9%),^4^, ^17^, ^26^, ^31^, ^32^, ^34^, ^35^, ^36^, ^37^, ^40^, ^42^, ^49^, ^54^, ^55^, ^58^ three outcomes by 11 reviews (23.4%)^6^, ^22^, ^25^, ^27^, ^33^, ^44^, ^45^, ^46^, ^50^, ^56^, ^60^ and 4–9 outcomes by eight reviews (17%).^19^, ^30^, ^41^, ^43^, ^48^, ^51^, ^57^, ^59^ Overall, the 47 eligible reviews reported data on 18 unique pooled outcome estimates including death in 36 reviews,^4^, ^6^, ^17^, ^18^, ^19^, ^22^, ^24^, ^25^, ^27^, ^30^, ^31^, ^32^, ^33^, ^34^, ^35^, ^36^, ^37^, ^38^, ^39^, ^41^, ^42^, ^43^, ^44^, ^45^, ^46^, ^47^, ^48^, ^49^, ^54^, ^55^, ^56^, ^58^, ^59^, ^60^ ICU admission in nine reviews,^27^, ^28^, ^30^, ^41^, ^43^, ^48^, ^51^, ^56^, ^59^ death/ICU admission as a composite outcome in 16 reviews,^4^, ^20^, ^21^, ^23^, ^26^, ^29^, ^31^, ^32^, ^40^, ^41^, ^43^, ^45^, ^51^, ^55^, ^59^ risk of acquiring COVID‐19 infection in 15 reviews,^19^, ^25^, ^27^, ^40^, ^41^, ^43^, ^44^ severe COVID‐19 infection in 22 reviews,^6^, ^17^, ^19^, ^22^, ^25^, ^30^, ^33^, ^34^, ^35^, ^36^, ^37^, ^41^, ^42^, ^43^, ^44^, ^45^, ^46^, ^48^, ^59^, ^60^ hospitalisation in nine reviews,^19^, ^30^, ^41^, ^43^, ^48^, ^59^ length of hospital stay in five reviews,^19^, ^22^, ^30^, ^46^, ^59^ use of mechanical ventilator in three reviews,^30^, ^41^ risk of severe acute respiratory syndrome (SARS) in two reviews,^26^, ^59^ and each of hospital discharge,^30^ ICU admission/mechanical ventilator use,^41^ risk of COVID‐19 infection/hospitalisation,^53^ severe pneumonia,^41^ level of serum creatinine,^57^ d‐dimer,^57^ cough,^57^ fever^57^ and renal dialysis^59^ in one review; accordingly, nine out of these 18 outcomes were included in the meta‐analysis as they were reported by at least two reviews. In terms of the exposure, ACEIs and ARBs were evaluated as one class (ACEIs/ARBs) in all the eligible 47 reviews apart from three,^26^, ^53^, ^57^ and as separate classes in 17^4^, ^6^, ^23^, ^25^, ^26^, ^27^, ^30^, ^31^, ^38^, ^40^, ^41^, ^43^, ^47^, ^50^, ^53^, ^54^, ^58^ and 16^4^, ^6^, ^23^, ^25^, ^26^, ^27^, ^30^, ^31^, ^38^, ^40^, ^41^, ^43^, ^50^, ^53^, ^54^, ^58^ reviews, respectively. The majority of the reviews (66%; *n* = 31) only evaluated one exposure, mainly ACEIs/ARBs combined as one class (*n* = 30); whereas one‐third of them (29.8%; *n* = 14) reported data for the three levels of exposure (ACEIs/ARBs, ACEIs and ARBs).

### 3.2 Degree of overlap between the 47 included reviews

An analysis of the degree of overlap of the studies within the included 47 reviews was conducted. However, data on the included studies were not fully reported by Zhang G (2020) et al (59), Zhang X (2020) et al (6) and Greco (2020) et al (34). In total, 168 studies were included within the 47 eligible reviews. Of these, the majority of studies (*n* = 99) were included in three or less reviews, with 71 of these included within only one review. The study included by most reviews was by Li J et al,^64^ which was included within 37 of the 47 reviews. An analysis of the degree of overlap across the systematic reviews using a citation matrix and the Corrected Cover Area (CCA) revealed a CCA value of 9.2 indicating a moderate degree of overlap.

### 3.3 Quality assessment

Overall confidence in the results was ‘moderate’ for 10 (21.3%) reviews,^19^, ^25^, ^26^, ^30^, ^37^, ^41^, ^42^, ^43^, ^56^, ^59^ ‘low’ for 15 (30.6%) reviews,^4^, ^5^, ^20^, ^21^, ^22^, ^27^, ^28^, ^31^, ^34^, ^45^, ^49^, ^50^, ^51^, ^55^, ^60^ and ‘critically low’ for 22 (44.9%) reviews^6^, ^17^, ^18^, ^23^, ^24^, ^29^, ^32^, ^33^, ^35^, ^36^, ^38^, ^39^, ^40^, ^44^, ^46^, ^47^, ^48^, ^52^, ^53^, ^54^, ^57^, ^58^ (Supplementary file S5). Considering the critical domains, most reviews were considered to have had a satisfactory technique for the statistical combination of results (*n* = 45, 95.7%)^4^, ^5^, ^6^, ^17^, ^18^, ^19^, ^20^, ^21^, ^22^, ^24^, ^25^, ^26^, ^27^, ^28^, ^29^, ^30^, ^31^, ^32^, ^33^, ^34^, ^35^, ^36^, ^37^, ^38^, ^39^, ^40^, ^41^, ^42^, ^43^, ^44^, ^45^, ^46^, ^47^, ^48^, ^49^, ^50^, ^51^, ^52^, ^53^, ^54^, ^55^, ^56^, ^57^, ^59^, ^60^ and for assessing risk of bias (*n* = 38, 80.1%).^4^, ^5^, ^6^, ^17^, ^19^, ^20^, ^21^, ^22^, ^23^, ^25^, ^26^, ^27^, ^28^, ^30^, ^31^, ^34^, ^35^, ^36^, ^37^, ^38^, ^40^, ^41^, ^42^, ^43^, ^44^, ^45^, ^46^, ^48^, ^49^, ^50^, ^51^, ^52^, ^53^, ^55^, ^56^, ^57^, ^59^, ^60^ Less reviews were favourably considered in terms of accounting for risk of bias when interpreting and discussing the results (*n* = 32, 68.1%), with appropriate conduct of publication bias (*n* = 33),^4^, ^5^, ^6^, ^17^, ^19^, ^20^, ^21^, ^23^, ^24^, ^25^, ^26^, ^27^, ^30^, ^31^, ^32^, ^33^, ^37^, ^38^, ^41^, ^42^, ^43^, ^44^, ^45^, ^47^, ^49^, ^50^, ^51^, ^53^, ^56^, ^57^, ^59^, ^60^ and only 15 (31.9%) reviews referred to the review methods being established a priori.^19^, ^22^, ^25^, ^26^, ^28^, ^30^, ^34^, ^37^, ^41^, ^42^, ^43^, ^52^, ^55^, ^56^, ^59^

### 3.4 Effect of ACEIs/AEBs (as a one group) on the study outcomes

**FIGURE 2:**
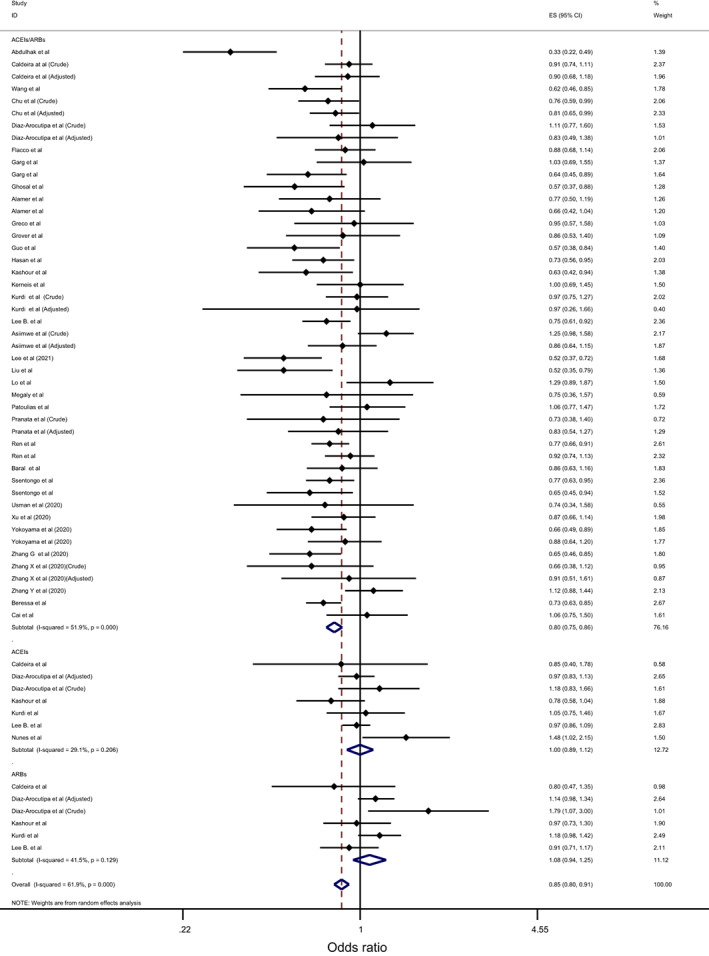
Forest plot depicting pooled estimates for the association between mortality and renin–angiotensin system drugs use

**FIGURE 3:**
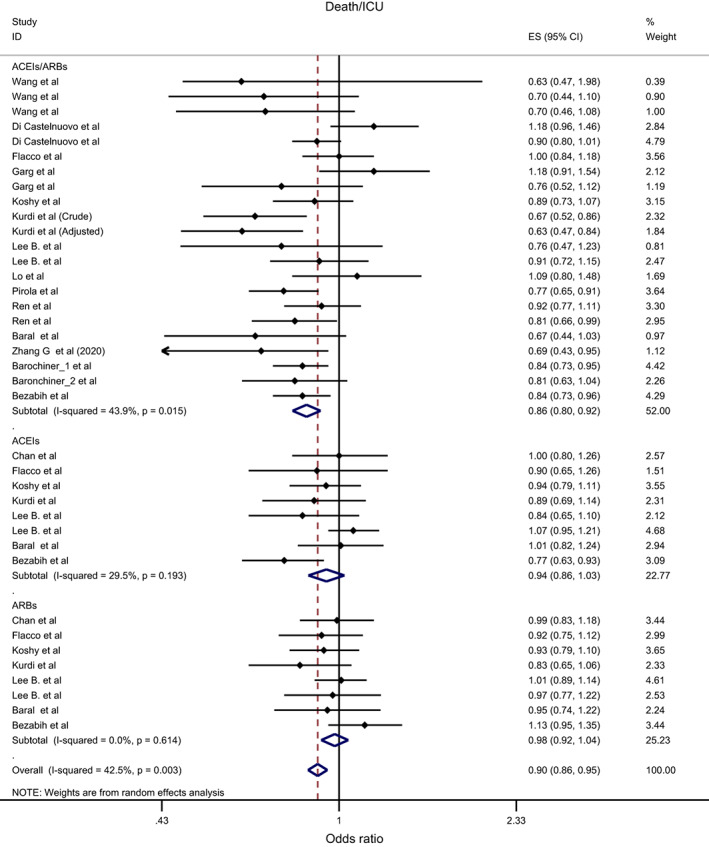
Forest plot depicting pooled estimates for the association between death/Intensive Care Unit (as a composite outcome) and renin–angiotensin system drugs use

**FIGURE 4:**
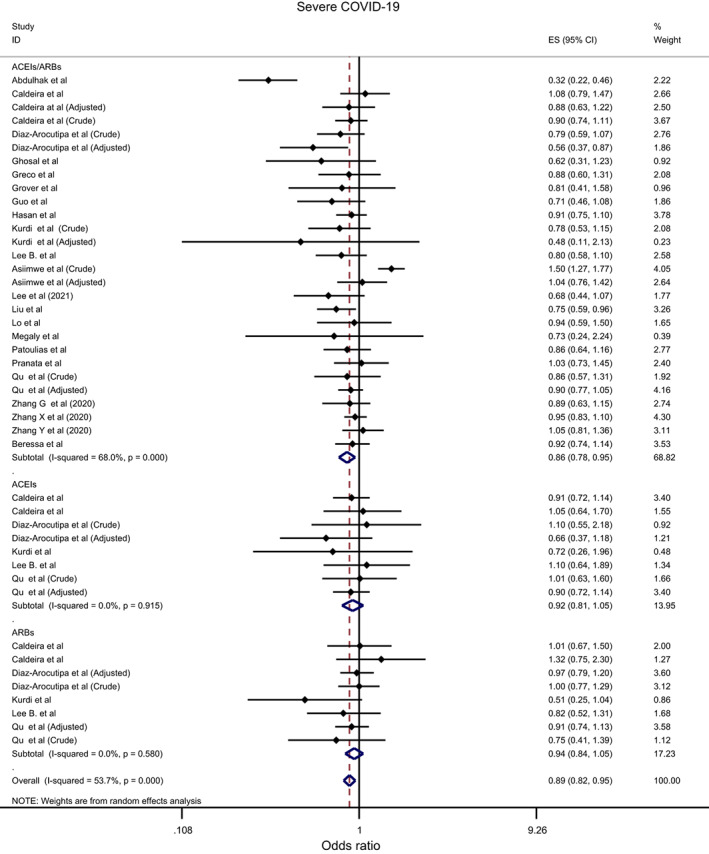
Forest plot depicting pooled estimates for the association between severe COVID‐19 infection and renin–angiotensin system drugs use

**FIGURE 5:**
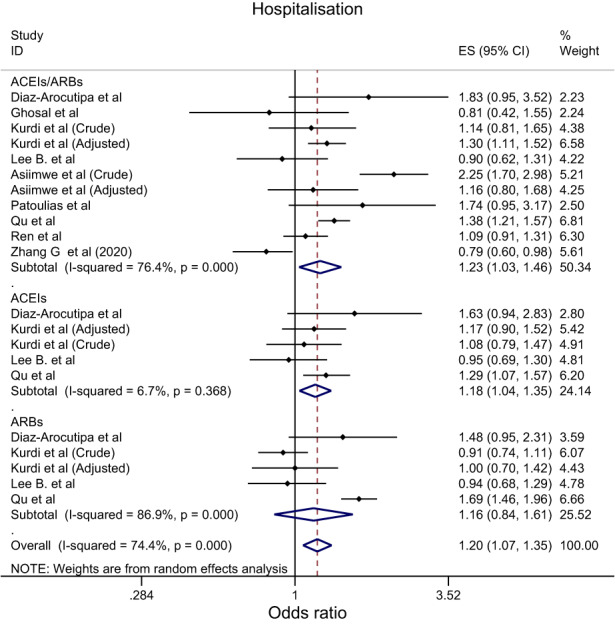
Forest plot depicting pooled estimates for the association between hospitalisation and renin–angiotensin system drugs use

**FIGURE 6:**
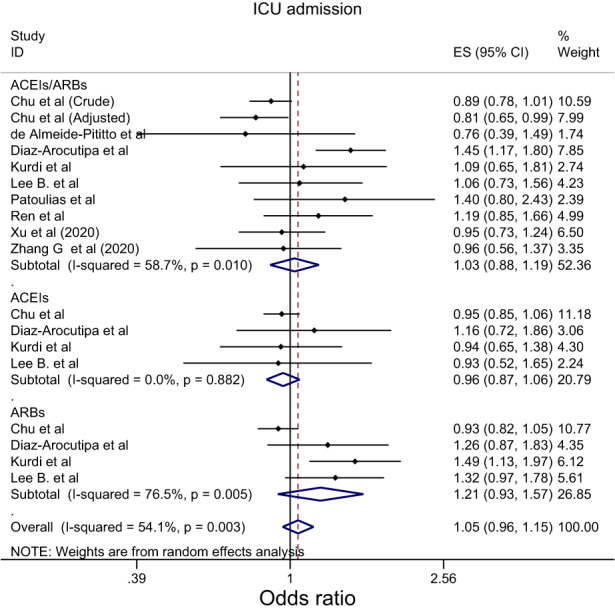
Forest plot depicting pooled estimates for the association between Intensive Care Unit admission and renin–angiotensin system drugs use

**FIGURE 7:**
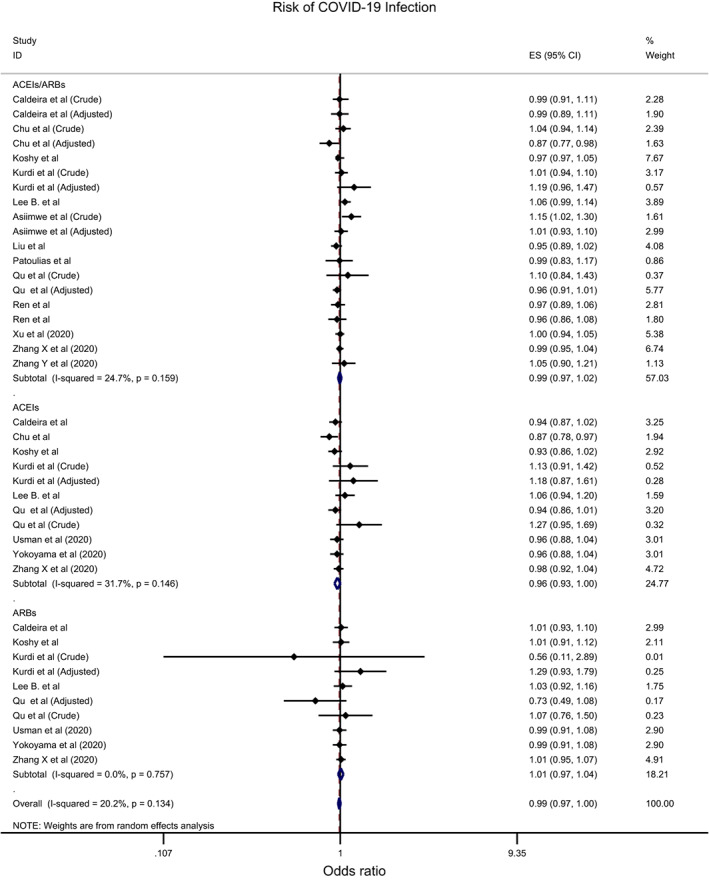
Forest plot depicting pooled estimates for the association between risk of acquiring COVID‐19 infection and renin–angiotensin system drugs use

**FIGURE 8:**
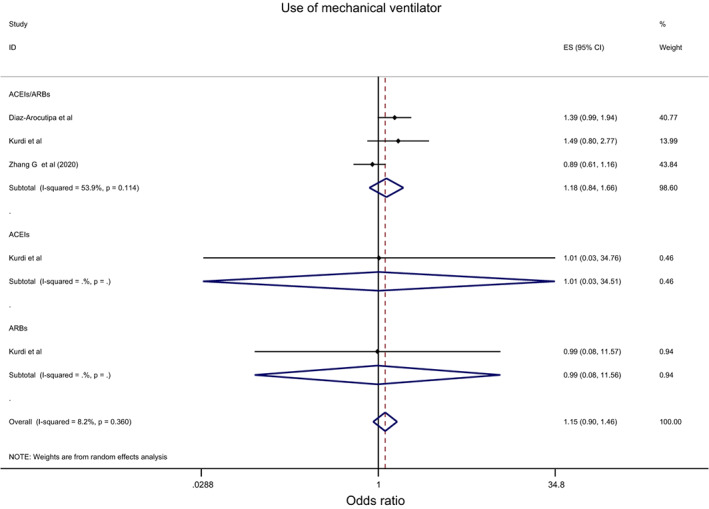
Forest plot depicting pooled estimate for the association between use of mechanical ventilator and renin–angiotensin system drugs use

**FIGURE 9:**
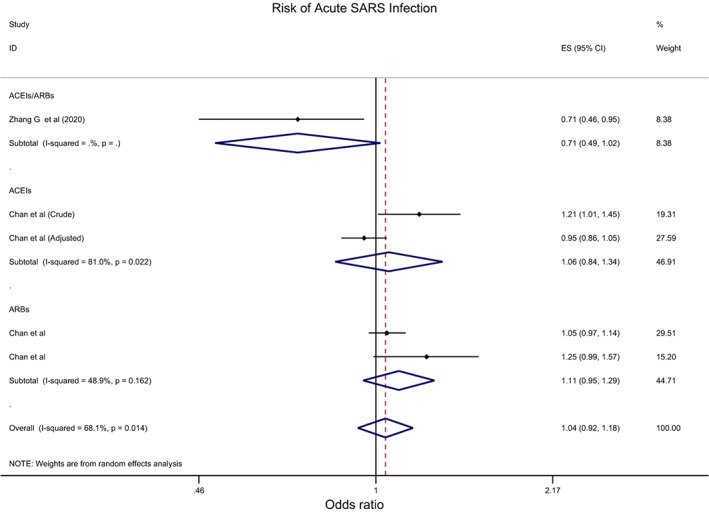
Forest plot depicting pooled estimates for the association between risk of severe acute respiratory syndrome (SARS) and renin–angiotensin system drugs use

**FIGURE 10:**
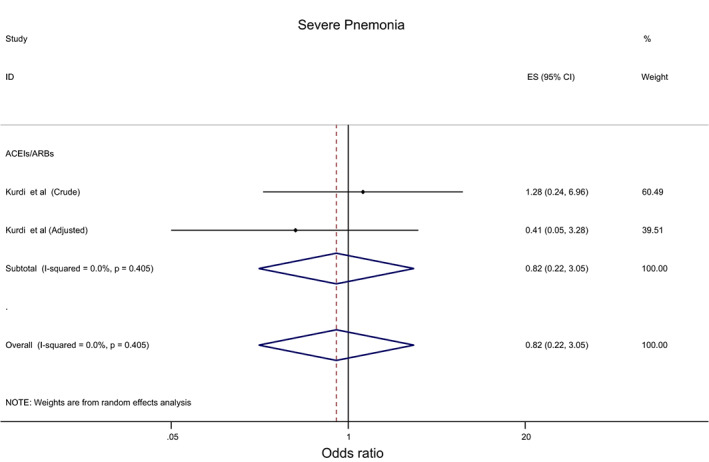
Forest plot depicting pooled estimates for the association between severe pneumonia and renin–angiotensin system drugs use

Overall, the effect of ACEIs/ARBs on nine COVID‐19‐related clinical outcomes was evaluated (Table 1). The combined pooled meta‐analysis estimates indicated that ACEIs/ARBs use was associated with a significant reduction in three clinical outcomes including death (OR = 0.80, 95%CI = 0.75–0.86; I^2^ = 51.9%) (Figure 2), death/ICU admission as composite outcome (OR = 0.86, 95%CI = 0.80–0.92; I2 = 43.9%) (Figure 3), and severe COVID‐19 infection (OR = 0.86, 95% CI = 0.78–0.95; I2 = 68%) (Figure 4); on the contrary, ACEIs/ARBs was associated with a significant increase in hospitalisation (OR = 1.23, 95%CI = 1.04–1.46; I2 = 76.4%) (Figure 5). However, there was insignificant association with each of ICU admission (Figure 6), risk of acquiring COVID‐19 infection (Figure 7), use of mechanical ventilator (Figure 8), risk of SARS (Figure 9), and risk of severe pneumonia (Figure 10).

**TABLE 1:** Meta‐analyses pooled estimates with 95%CI of the effects of ACEIs/ARBs on COVID‐19‐related clinical outcomes

Outcomes	ACEIs/ARBs	*p*‐value	ACEIs	*p*‐value	ARBs	*p*‐value
**Death**	0.80 (0.75, 0.86)	<0.001	0.91 (0.89, 1.12)	0.984	1.10 (0.94, 1.25)	0.263
Number of studies	47		7		6	
I‐squared	51.9%	0.001	29.1%	0.206	41.5%	0.129
**ICU**	1.03 (0.86, 1.19)	0.721	0.96 (0.87, 1.1)	0.406	1.21 (0.93, 1.47)	0.312
Number of studies	10		4		4	
I‐squared (*p*‐value)	58.7%	0.01	0%	0.882	76.5%	0.005
**Death/ICU**	0.86 (0.80, 0.92)	<0.001	0.94 (0.86, 1.03)	0.167	0.98 (0.92, 1.05)	0.530
Number of studies	22		8		8	
I‐squared (*p*‐value)	43.9%	0.015	29.5%	0.193	0%	0.614
**Risk of COVID‐19**	0.99 (0.97, 1.02)	0.560	0.97 (0.93, 1.01)	0.058	1.01 (0.97, 1.04)	0.726
Number of studies	19		11		10	
I‐squared (*p*‐value)	24.7%	0.159	31.7%	0.146	0%	0.757
**Severe COVID‐19**	0.86 (0.78, 0.95)	0.003	0.92 (0.81, 1.05)	0.232	0.94 (0.84, 1.05)	0.281
Number of studies	28		8		8	
I‐squared (*p*‐value)	68%	<0.001	0%	0.951	53.7%	0.580
**Severe pneumonia**	0.82 (0.22, 3.05)	0.765	NA		NA	
Number of studies	2					
I‐squared (*p*‐value)	0%	0.405				
**Hospitalisation**	1.23 (1.04, 1.46)	0.019	1.18 (1.04, 1.35)	0.012	1.17 (0.84, 1.61)	0.354
Number of studies	11		5		5	
I‐squared (*p*‐value)	76.4%	<0.001	6.7%	0.368	86.9%	<0.001
**Ventilator use**	1.18 (0.84, 1.66)	0.347	1.01 (0.03, 34.52)	0.994	0.985 (0.084, 11.57)	0.990
Number of studies	3		1		1	
I‐squared (*p*‐value)	53.9%	0.114	NA		NA	
**Acute SARS infection**	0.71 (0.49, 1.02)	0.064	1.06 (0.84, 1.34)	0.633	1.11 (0.95, 1.29)	0.493
Number of studies	1		2		2	
I‐squared (*p*‐value)	NA		81%	0.022	48.9%	0.162

Abbreviation: NA, not applicable indicating not enough studies to perform meta‐analyses.

However, the subgroup analyses indicated different results for some of the outcomes (Table 2). Firstly, despite the consistent significant reduction in death in association with ACEIs/ARBs use regardless of studies' crude/adjusted measure of effects, peer‐review status and hypertension use status, there was a trend towards lower protective effective of ACEIs/ARBs on death as the quality of the studies enhanced from critically low (OR = 0.75, 95%CI = 0.66–0.85; I^2^ = 60.4%) to moderate (OR = 0.85, 95%CI = 0.75–0.96; I^2^ = 53.4%) (Supplementary file S6A; Table 2). Similarly, the significant reduction in death/ICU admission associated with ACEIs/ARBs appeared to be higher among the studies, which presented adjusted measure of effects (adjusted: OR = 0.63, 95%CI = 0.47–0.84 vs. crude: OR = 0.87, 95%CI = 0.81–0.93); and the pooled estimates for association ranged from insignificant association among the critically low‐quality studies (OR = 0.94, 95%CI = 0.84–1.06; I^2^ = 57.4%) to a significantly higher reduction among the moderate‐quality studies (OR = 0.74, 95%CI = 0.63–0.85; I^2^ = 18.9%); (Supplementary file S7A; Table 2). Besides, the significant protective impact of ACEIs/ARBs on death/ICU admission was observed only among peer‐reviewed studies (peer‐reviewed: OR = 0.85, 95%CI = 0.79–0.92 vs. non‐peer‐reviewed: OR = 0.89, 95%CI = 0.75–1.10) and studies including hypertensive patients (OR = 0.85, 95%CI = 0.80–0.90) Supplementary file S7A; Table 2).

**TABLE 2:** Subgroup meta‐analyses pooled estimates with 95%CI of the effects of ACEIs/ARBs on COVID‐19‐related clinical outcomes

	ACEIs/ARBs	ACEIs	ARBs
	**Death (*n* = 60)**		
**Adjusted outcome measure**			
Adjusted OR	0.80 (0.74, 0.91)	0.90 (0.89, 1.12)	1.1 (0.96, 1.26)
Crude OR	0.80 (0.73, 0.86)	1.10 (0.92, 1.25)	1.1 (0.85, 1.42)
Number of studies	10 vs. 37	2 vs. 5	2 vs. 4
I‐squared (*p*‐value)	0.0% (0.947) vs. 61% (<0.001)	40.3% (0.196) vs. 26.7% (0.244)	0.0% (0.335) vs. 60.6% (0.055)
**Peer‐reviewed article?**			
Yes	0.80 (0.76, 0.85)	1.0 (0.83, 1.2)	1.02 (0.87, 1.19)
No	0.79 (0.66, 0.95)	1.0 (0.87, 1.16)	1.33 (0.88, 2.03)
Number of studies	33 vs. 14	5 vs. 2	4 vs. 2
I‐squared (*p*‐value)	25.3% (0.095) vs. 75.3% (>0.001)	45.7% (0.117) vs. 2.5% (0.331)	27.2% (0.249) vs. 62.9% (0.101)
**Study's quality**			
Critically low	0.75 (0.66, 0.85)	1.06 (0.57, 1.99)	0.97 (0.37, 1.29)
Low	0.81 (.075, 0.88)	NA	NA
Moderate	0.85 (0.75, 0.96)	0.99 (0.90, 1.10)	1.11 (0.94, 1.30)
Number of studies	21 vs. 12 vs. 14	2 vs. 0 vs. 5	1 vs. 0 vs. 5
I‐squared (*p*‐value)	60.4% (>0.001) vs. 18.8% (0.259) vs. 53.4% (0.009)	85.8% (0.008) vs. NA vs. 29.1% (0.206)	NA vs. NA vs. 48.4% (0.101)
**Hypertension use status**			
Hypertensive patients	0.74 (0.69, 0.79)	0.97 (0.86, 1.09)	0.91 (0.71, 1.17)
Not recorded	0.84 (0.77, 0.92)	1.02 (0.87, 1.21)	1.13 (0.98, 1.31)
Number of studies	15 vs. 32	1 vs. 6	1 vs. 5
I‐squared (*p*‐value)	0.0% (0.617) vs. 57.3% (>0.001)	NA vs. 39.9% (0.140)	NA vs. 33.5% (0.129)
	**ICU admission (*n* = 18)**		
**Adjusted outcome measure**			
Adjusted OR	0.86 (0.73, 1.02)	NA	NA
Crude OR	1.09 (0.91, 1.32)	0.96 (0.87, 1.06)*	1.21 (0.93, 1.57)*
Number of studies	2 vs. 8	0 vs. 4	0 vs. 4
I‐squared (*p*‐value)	0.0% (0.356) vs. 59.8% (0.015)	NA vs. 0.0% (0.882)	NA vs. 76.5% (0.005)
**Peer‐reviewed article?**			
Yes	0.93 (0.85, 1.01)	0.95 (0.86, 1.05)	1.20 (0.87, 1.66)
No	1.45 (1.17, 1.80)	1.16 (0.72, 1.86)	1.26 (0.87, 1.83)
Number of studies	9 vs. 1	3 vs. 1	3 vs. 1
I‐squared (*p*‐value)	0.0% (0.488) vs. NA	0.0% (0.997) vs. NA	83.1% (0.003) vs. NA
**Study's quality**			
Critically low	1.40 (0.80, 2.44)	NA	NA
Low	0.90 (0.78, 1.03)	0.95 (0.85, 1.06)	0.93 (0.82, 1.05)
Moderate	1.12 (0.92, 1.37)	1.0 (0.77, 1.30)	1.37 (1.15, 1.64)
Number of studies	1 vs. 4 vs. 5	0 vs. 1 vs. 3	0 vs. 1 vs. 3
I‐squared (*p*‐value)	NA vs. 22.6% (0.275) vs. 45% (0.122)	NA vs. NA vs. 0.0% (0.770)	NA vs. NA vs. 0.0% (0.742)
**Hypertension use status**			
Hypertensive patients	0.97 (0.75, 1.27)	0.93 (0.52, 1.66)	1.32 (0.97, 1.79)
Not recorded	1.05, 0.87, 1.27)	0.96 (0.87, 1.06)	1.18 (0.85, 1.64)
Number of studies	3 vs. 7	1 vs. 3	1 vs. 3
I‐squared (*p*‐value)	0.0% (0.697) vs. 71.5% (0.002)	NA vs. 0.0% (0.722)	NA vs. 80.8% (0.006)
	**Death/ICU admission (*n* = 38)**		
**Adjusted outcome measure**			
Adjusted OR	0.63 (0.47, 0.84)	1.0 (0.80, 1.26)	1.0 (0.83, 1.18)
Crude OR	0.87 (0.81, 0.93)	0.93 (0.85, 1.03)	0.98 (0.91, 1.05)
Number of studies	1 vs. 21	1 vs. 7	1 vs. 7
I‐squared (*p*‐value)	NA vs. 38.9% (0.036)	NA vs. 38.5% (0.135)	NA vs. 0.0% (0.498)
**Peer‐reviewed article?**			
Yes	0.85 (0.79, 0.92)	0.99 (0.92, 1.10)	0.96 (0.89, 1.03)
No	0.89 (0.75, 1.10)	0.77 (0.63, 0.94)	1.13 (0.95, 1.34)
Number of studies	18 vs. 4	7 vs. 1	7 vs. 1
I‐squared (*p*‐value)	45.5% (0.019) vs. 51.5% (0.103)	0.0% (0.605) vs. NA	0.0% (0.874) vs. NA
**Study's quality**			
Critically low	0.94 (0.84, 1.06)	0.86 (0.70, 1.04)	1.02 (0.85, 1.24)
Low	0.85 (0.79, 0.92)	0.98 (0.82, 1.16)	0.93 (0.80, 1.10)
Moderate	0.74 (0.63, 0.85)	0.99 90.88, 1.10)	0.98 (0.89, 1.06)
Number of studies	6 vs. 11. vs. 5	2 vs. 2 vs. 4	2 vs. 2 vs. 4
I‐squared (*p*‐value)	57.4% (0.038) vs. 15.8% (0.293) vs. 18.9% (0.294)	56.3% (0.130) vs. 0.0% (0.568) vs. 20.7% (0.286)	60% (0.114) vs. 0.0% (0.865) vs. 0.0% (0.572)
**Hypertension use status**			
Hypertensive patients	0.85 (0.80, 0.9)	0.9 (0.75, 1.08)	1.01 (0.93, 1.10)
Not recorded	0.88 (0.76, 1.03)	0.96 (0.87, 1.06)	0.93 (0.85, 1.03)
Number of studies	13 vs. 9	4 vs. 4	4 vs. 4
I‐squared (*p*‐value)	0.0% (0.595) vs. 69% (0.001)	67.1% (0.028) vs. 0.0% (0.852)	0.0% (0.473) vs. 0.0% (0.723)
	**Risk of COVID‐19 infection (*n* = 40)**		
**Adjusted outcome measure**			
Adjusted OR	0.98 (0.94, 1.03)	1.0 (0.82, 1.2)	0.98 (0.56, 1.7)
Crude OR	1.0 (0.97, 1.02)	0.97 (0.93, 1.01)	1.0 (0.97, 1.04)
Number of studies	6 vs. 13	2 vs. 9	2 vs. 8
I‐squared (*p*‐value)	41.7% (0.127) vs. 18.7% (0.255)	49% (0.161) vs. 36.6% (0.125)	78.9% (0.03) vs. 0.0% (0.993)
**Peer‐reviewed article?**			
Yes	0.99 (0.97, 1.01)	0.96 (0.92, 1.01)	1.01 (0.98, 1.05)
No	1.03 (0.96, 1.10)	0.97 (0.89, 1.10)	0.97 (0.85, 1.11)
Number of studies	14 vs. 5	8 vs. 3	7 vs. 3
I‐squared (*p*‐value)	14.6% (0.294) vs. 52.5% (0.077)	34.8% (0.150) vs. 48.6% (0.143)	0.0% (0.814) vs. 18.1% (0.295)
**Study's quality**			
Critically low	0.97 (0.95, 1.0)	0.96 (0.93, 0.99)	1.0 (0.96, 1.04)
Low	0.97 (0.93, 1.01)	0.95 (0.84, 1.09)	0.90 (0.62, 1.30)
Moderate	1.03 (0.99, 1.06)	1.03 (0.93, 1.14)	1.03 (0.96, 1.10)
Number of studies	4 vs. 7 vs. 8	4 vs. 3 vs. 4	4 vs. 2 vs. 4
I‐squared (*p*‐value)	0.0% (0.780) vs. 17.5% (0.296) vs. 12.7% (0.331)	0.0% (0.811) vs. 66.7% (0.050) vs. 45.3% (0.140)	0.0% (0.970) vs. 51.6% (0.151) vs. 0.0% (0.467)
**Hypertension use status**			
Hypertensive patients	1.02 (0.93, 1.11)	1.0 (0.91, 1.11)	1.0 (0.94, 1.08)
Not recorded	0.99 (0.97, 1.01)	0.96 (0.92, 0.99)	1.0 (0.97, 1.05)
Number of studies	2 vs. 17	2 vs. 9	2 vs. 8
I‐squared (*p*‐value)	58.3% (0.122) vs. 19.7% (0.224)	42.0% (0.189) vs. 33.5% (0.150)	0.0% (0.590) vs. 0.0% (0.595)
	**Severe COVID‐19 (*n* = 44)**		
**Adjusted outcome measure**			
Adjusted OR	0.88 (0.78, 0.99)	0.86 (0.70, 1.07)	0.94 (0.81, 1.10)
Crude OR	0.86 (0.75, 0.97)	0.96 (0.81, 1.14)	0.93 (0.78, 1.13)
Number of studies	6 vs. 22	2 vs. 6	2 vs. 6
I‐squared (*p*‐value)	19.3% (0.287) vs. 73% (>0.001)	0.0% (0.330) vs. 0.0% (0.954)	0.0% (0.674) vs. 8.8% (0.360)
**Peer‐reviewed article?**			
Yes	0.89 (0.83, 0.96)	0.94 (0.78, 1.14)	0.91 (0.66, 1.25)
No	0.82 (0.66, 1.01)	0.9 (0.75, 1.10)	0.95 (0.83, 1.10)
Number of studies	15 vs. 13	4 vs. 4	4 vs. 4
I‐squared (*p*‐value)	0.0% (0885) vs. 84% (>0.001)	0.0% (0.832) vs. 0.0% (0.646)	36.3% (0.194) vs. 0.0% (0.821)
**Study's quality**			
Critically low	0.69 (0.53, 0.92)	NA	NA
Low	0.93 (0.85, 1.03)	0.92 (0.75, 1.31)	0.89 (0.73, 1.09)
Moderate	0.89 (0.77, 1.04)	0.92 (0.78, 1.10)	0.96 (0.84, 1.10)
Number of studies	7 vs. 7 vs. 14	0 vs. 2 vs. 6	0 vs. 2 vs. 6
I‐squared (*p*‐value)	80.5% (>0.001) vs. 0.0% (0.954) vs. 69.8% (>0.001)	NA vs. 0.0% (0.664) vs. 0.0% (0.782)	NA vs. 0.0% (0.557) vs. 0.0% (0.426)
**Hypertension use status**			
Hypertensive patients	0.89 (0.77, 1.01)	1.10 (0.64, 1.89)	0.82 (0.52, 1.30)
Not recorded	0.85 (0.758, 0.96)	0.91 (0.79, 1.10)	0.95 (0.84, 1.10)
Number of studies	5 vs. 23	1 vs. 7	1 vs. 7
I‐squared (*p*‐value)	0.0% (0.684) vs. 73.1% (>0.001)	NA vs. 0.0% (0.899)	Na vs. 0.0% (0.506)
	**Hospitalisation (*n* = 21)**		
**Adjusted outcome measure**			
Adjusted OR	1.33 (1.21, 1.47)	1.25 (1.10, 1.46)	1.33 (0.80, 2.23)
Crude OR	1.21 (0.91, 1.61)	1.10 (0.86, 1.41)	1.02 (0.79, 1.31)
Number of studies	3 vs. 8	2 vs. 3	2 vs. 3
I‐squared (*p*‐value)	0.0% (0.634) vs. 81.5% (>0.001)	0.0% (0.556) vs. 27.9% (0.250)	86.1% (0.007) vs. 49% (0.141)
**Peer‐reviewed article?**			
Yes	1.11 (0.90, 1.31)	1.11 (0.91, 1.27)	0.93 (0.80, 1.10)
No	1.45 (1.10, 2.0)	1.32 (1.10, 1.59)	1.67 (1.45, 1.92)
Number of studies	6 vs. 5	3 vs. 2	3 vs. 2
I‐squared (*p*‐value)	66.2% (0.011) vs. 73.1% (0.005)	0.0% (0.611) vs. 0.0% (0.432)	0.0% (894) vs. 0.0% (0.578)
**Study's quality**			
Critically low	1.20 (0.57, 2.54)	NA	NA
Low	1.24 (0.98, 1.56)	1.29 (1.07, 1.56)	1.69 (1.46, 1.96)
Moderate	1.24 (0.94, 1.63)	1.12 (0.95, 1.31)	0.99 (0.94, 1.19)
Number of studies	2 vs. 2 vs. 7	0 vs. 1 vs. 4	0 vs. 1 vs. 4
I‐squared (*p*‐value)	64.8% (0.092) vs. 76.5% (0.039) vs. 82.9% (>0.001)	NA vs. NA vs. 0.0% (0.368)	NA vs. NA vs. 23.9% (0.268)
**Hypertension use status**			
Hypertensive patients	0.82 (0.67, 1.01)	0.95 (0.69, 1.30)	0.94 (0.68, 1.31)
Not recorded	1.35 (1.15, 1.58)	1.23 (1.10, 1.41)	1.23 (0.84, 1.78)
Number of studies	2 vs. 9	1 vs. 4	1 vs. 4
I‐squared (*p*‐value)	0.0% (0.568) vs. 66% (0.003)	NA vs. 0.0% (0.553)	NA vs. 88.7% (>0.001)
	**Ventilator use (*n* = 5)**		
**Adjusted outcome measure**			
Adjusted OR	NA	NA	NA
Crude OR	1.18 (0.84, 1.66)*	1.01 (0.03, 34.52)*	0.985 (0.084, 11.57)*
Number of studies	0 vs. 3	0 vs. 1	0 vs. 1
I‐squared (*p*‐value)	NA vs. 53.4% (0.114)	NA	NA
**Peer‐reviewed article?**			
Yes	1.10 (0.66, 1.75)	1.01 (0.03, 34.52)*	0.985 (0.084, 11.57)*
No	1.39 (0.99, 1.95)	NA	NA
Number of studies	2 vs. 1	1 vs. 0	1 vs. 0
I‐squared (*p*‐value)	52.6% (0.146) vs. NA	NA	NA
**Study's quality**			
Critically low	NA	NA	NA
Low	NA	NA	NA
Moderate	1.18 (0.84, 1.66)*	1.01 (0.03, 34.52)*	0.985 (0.084, 11.57)*
Number of studies	0 vs. 0 vs. 3	0 vs. 0 vs. 1	0 vs. 0 vs. 1
I‐squared (*p*‐value)	NA vs. NA vs. 53.4% (0.114)	NA	NA
**Hypertension use status**			
Hypertensive patients	0.89 (0.65, 1.23)	NA	NA
Not recorded	1.41 (1.10, 1.90)	1.014 (0.030, 34.758)*	0.985 (0.084, 11.570)*
Number of studies	1 vs. 2	0 vs. 1	0 vs. 1
I‐squared (*p*‐value)	NA vs. 0.0% (0.844)	NA	NA
Acute SARS (n = 5)			
**Adjusted outcome** measure			
Adjusted OR	NA	0.95 (0.86, 1.05)	1.05 (0.97, 1.14)
Crude OR	0.71 (0.49, 1.02)	1.21 (1.01, 1.45)	1.25 (0.99, 1.57)
Number of studies	0 vs. 1	1 vs. 1	1 vs. 1
I‐squared (*p*‐value)	NA	NA	NA
**Peer‐reviewed article?**			
Yes	0.71 (0.49, 1.02)*	1.06 (0.84, 1.34)*	1.11 (0.95, 1.29)*
No	NA	NA	NA
Number of studies	1 vs. 0	2 vs. 0	2 vs. 0
I‐squared (*p*‐value)	NA	81% (0.022) vs. NA	48.9% (0.162) vs. NA
**Study's quality**			
Critically low			
Low	NA	NA	NA
Moderate	NA	NA	NA
Number of studies	0.71 (0.49, 1.02)	1.06 (0.84, 1.34)*	1.11 (0.95, 1.29)*
I‐squared (*p*‐value)	0 vs. 0 vs. 1	0 vs. 0 vs. 2	0 vs. 0 vs. 2
Hypertension use status		NA vs. NA. vs. 81% (0.022)	NA vs. NA. vs. 48.9% (0.162)
**Hypertensive patients**	0.71 (0.49, 1.02)	NA	NA
Not recorded	NA	1.06 (0.84, 1.34)	1.11 (0.95, 1.29)
Number of studies	1 vs. 0	0 vs. 2	0 vs. 2
I‐squared (*p*‐value)	NA	NA vs. 81% (0.022)	NA vs. 48.9% (0.162)

*Note*: *Indicates that the pooled estimate is the same as the overall analyses because all the studies were in one group.

Abbreviation: NA, not applicable indicating that no studies were available to perform meta‐analyses for these outcomes.

Likewise, the protective effect of ACEIs/ARBs use on severe COVID‐19 infection was observed only among: peer‐reviewed studies (peer‐reviewed: OR = 0.89, 95%CI = 0.83–0.96 vs. non‐peer‐reviewed: OR = 0.82, 95%CI = 0.66–1.01), studies that did not record the hypertension status of their patients (OR = 0.85, 95%CI = 0.76–0.96), and critically low‐quality studies (OR = 0.69, 95%CI = 0.53–0.92); and in fact the protective effect disappeared completely as the quality of the studies improved since insignificant association was observed among both low‐ and moderate‐quality studies (OR = 0.93, 95%CI = 0.85–1.03; OR = 0.89, 95%CI = 0.77–1.04, respectively) (Supplementary file S8A; Table 2 **)**. In terms of ACEIs/ARBs' increasing impact on hospitalisation, this impact was demonstrated only among the studies which presented adjusted measure of effects (adjusted: OR = 1.33, 95%CI = 1.21–1.47 vs. crude: OR = 1.21, 95%CI = 0.91–1.61), were not peer‐reviewed (OR = 1.45, 95%CI = 1.10–10.20 vs. peer‐reviewed: OR = 1.11, 95%CI = 0.90–1.31), and did not record the hypertension status of their patients (OR = 1.35, 95%CI = 1.15–1.58) (Supplementary file S9A; Table 2).

### 3.5 Effect of ACEIs and AEBs (as a separate group) on the study outcomes

Overall, the effect of ACEIs and ARBs on seven COVID‐19‐related clinical outcomes (death, ICU admission, death/ICU admission, risk of acquiring COVID‐19 infection, severe COVID‐19 infection, hospitalisation and acute SARS) was evaluated. Neither ACEIs nor ARBs had any significant impact on any of the seven studied outcomes (Figures 2, 3, 4, 5, 6, 7, 8, 9, 10, Table 1) except for hospitalisation whereby ACEIs use was associated with a significant increase in COVID‐19‐related hospitalisation (OR = 1.18, 95%CI = 1.04–1.35; I^2^ = 6.7%) (Figure 5; Table 1). These results were mostly consistent across all the subgroup analyses (Supplementary file S6B,C, 7B,C, 8B,C; Table 2) except for the increasing effect of ACEIs on hospitalisation, which was only observed among those studies which did not record the hypertension status of their patients (OR = 1.23, 95%CI = 1.10–1.41) (Supplementary file S9B,C; Table 2). Results from the sensitivity analysis of adopting a lower significance threshold of <0.02 were consistent with those obtained from using the <0.05 significance threshold, indicating no effect of type I error on our pooled estimates' significance level.

### 3.6 Publication bias

Results from the funnel plots and Egger's asymmetry tests for the six outcomes that were reported by at least 10 studies indicated no evidence of significant publication bias in all of them except for death/ICU admission and severe COVID‐19 infection (*p*‐value = 0.022 and 0.019, respectively) (Supplementary file S10).

### 3.7 Influential analyses

The results from the influential analyses indicated that none of the combined pooled meta‐analysis estimates for the nine outcomes were dominated/influenced by an individual review/meta‐analysis since the omission of any of these individual reviews/meta‐analyses one at a time made no difference to the pooled meta‐analysis estimate because all of pooled meta‐analysis estimates were overlapping (Supplementary file S11). It is worth mentioning that it might be possible that one or few of the original primary studies influenced some of the 47 review/meta‐analyses included in our umbrella review but it was not possible for us to assess this information because these data were not reported by the included review/meta‐analyses.

## 4 DISCUSSION

This umbrella review for the first time combined all the available evidence so far from observational studies on the impact of ACEIs/ARBs on COVID‐19 clinical outcomes (47 systematic review studies which reported 213 meta‐analyses) into one pooled estimate. The collective, combined pooled estimates indicated evidence of statistically significant reduction in mortality, death/ICU admission, and severe COVID‐19 infection in association with ACEIs/ARBs use, but significant increase in the risk of hospitalisation (Table 1). Interestingly, there was no evidence of any significant association between ACEIs or ARBs and any of the nine COVID‐19‐related clinical outcomes analysed in our study.

Although the magnitude of observed impact of ACEIs/ARBs use on reducing mortality was decreasing as the quality of studies improved (Table 2), the evidence was overall mostly consistent across all the subgroup analyses including a greater impact among studies that included hypertensive patients compared with studies that did not record the hypertension status of their study population (Table 2). In terms of death/ICU admission, the quality of the evidence was even better because the impact of ACEIs/ARBs use was greater and significant only among moderate‐quality studies, peer‐reviewed studies and studies with hypertensive patients; however, the impact was significant regardless of whether the measure of effects was crude or adjusted, even though the impact was greater among studies with adjusted measure of effects compared those studies with crude measure of effects (Table 2). By contrast, the quality of the evidence for the impact of ACEIs/ARBs use on severe COVID‐19 was low since the significant reduction was only observed among critically low‐quality studies and, in fact, the significant association disappeared as the quality of the studied enhanced from critically low quality to either low or moderate quality (Table 2).

In terms of the impact of ACEIs/ARBs on hospitalisation, the quality of the evidence was low because the significant association was not apparent when the data were analysed by the quality of the studies, even though the magnitude of the effect was almost consistent across the various quality of the studies; besides, the significant increase in hospitalisation was observed only among studies that reported adjusted measure of effects, non‐peer‐reviewed studies and studies that did not record the hypertensive status of their study population (Table 2).

The subgroup analyses demonstrated low‐quality evidence regarding the different impact of ACEIs and ARBs (as separate groups) (Table 2). This observed difference has been suggested to be due to the increased level of angiotensin II, which occurs following ARBs treatment but not ACEIs, which in turn imposes an increased substrate load on ACE2 enzyme requiring its upregulation,^65^ hence facilitating COVID‐19 virus cell entry and its subsequent infectivity/pathogenicity.^66^ Furthermore, the increase in ACE2 activity demonstrated in patients with hypertension, either due to the pathophysiology of hypertension itself^67^ or the administration of ACEIs/ARBs as antihypertensive medications,^68^ could at least partially explain some of our study findings as why ACEIs/ARBs had significant greater impact on certain COVID‐19 clinical outcomes (i.e. mortality and death/ICU admission) only among studies that included patient with hypertension.

Several hypotheses (related to the pathophysiology of COVID‐19 infection and functions of ACE2) can explain the observed impact of ACEIs/ARBs in our current study. The adverse negative effects of ACEIs/ARBs could be due to ACEIs/ARBs ability to cause upregulation of ACE2 expression (the cell entry point for COVID‐19), hence facilitating and enhance COVID‐19 viral binding and cell entry,^68^ whereas the positive protective effects could be through ACEIs/ARBs blockage of the harmful angiotensin II‐ AT_1_R axis and their effects on angiotensin II expression leading to subsequent increase in the level of the protective angiotensin 1–7 and 1–9 which have anti‐inflammatory and vasodilatory effects (i.e. the corresponding increase in Ang I and Ang II in response to ACEIs and ARBs, respectively, use would activate and increase the protective Ang 1–7/1–9 axis via MasR and AT_2_R receptor resulting in their antioxidant, anti‐inflammatory, vasodilation and antifibrosis effects; also the protective Ang 1–7 level can be generated by neutral endopeptidase), hence potentially attenuating the cardiac and pulmonary damages of COVID‐19,^2^ which could potentially explain why discontinuation of ACEIs/ARBs might increase the risk of mortality due to the loss of this protective Ang 1–7/1–9 axis via MasR and AT_2_R pathway. Interestingly, a recent study has demonstrated that these benefits from ACEIs/ARBs antihypertensive drugs on in‐hospital mortality could be observed/extended with/to any first‐line antihypertensive drug treatment (OR: 0.25, 95%CI: 0.2–0.3)^69^; this is maybe due to the fact that almost all antihypertensive drugs are protective for the endothelium, an arterial layer targeted by COVID‐19. Genetic ACE2 polymorphism among some individuals has been also suggested as potential factor explaining, at least partially, the harmful effects on ACEIs/ARBs on COVID‐19 outcomes.^70^

It is worth to highlight that our study findings are still important despite the recently published randomised clinical trial (RCT)^71^ which found insignificant differences in the mean number of days alive/out of the hospital between those assigned to discontinue vs continue ACEIs or ARBs. This is because of certain points that are related to the findings from this RCT. First, this RCT was designed to evaluate the impact of continuing ACEIs or ARBs vs. their discontinuation after contracting COVID‐19 rather than evaluating ACEIs/ARBs use vs. nonuse of these medications which was the focus of most of the observational studies involved in our current study. Secondly, the RCT included only patients with mild or moderate COVID‐19 with more than half of the participants (57%; *n* = 376) having mild COVID‐19 and evaluated only two COVID‐19‐related clinical outcomes, namely days alive (mortality) and out‐of‐hospital days, hence leaving a big gap in the evidence around ACEIs/ARBs' impact on other important COVID‐19 clinical outcomes as well as limiting generalisability to patients with severe COVID‐19. Furthermore, although the RCT's participants were all hypertensive patients, about one‐third (~31%) and ~ 1% had diabetes and heart failure, respectively, which further limits the generalisability of the RCT's findings to these conditions for which ACEIs/ARBs are commonly indicated. Moreover, the RCT's participants were all from Brazil and hence extending the findings to other races or ethnicities will be limited; this is particularly important because there are evidence demonstrating that there are potential genetic variants of renin, angiotensinogen, ACE, angiotensin II and ACE2 among various populations that influence the function of the renin–angiotensin–aldosterone system, hence affecting someone’s response to the COVID‐19 infection.^72^ On the contrary, another more recent RCT^73^ exploring the effect of ACEIs/ARBs discontinuation vs. their continuation among 659 patients found that continuing ACEIs/ARBs in comparison with their discontinuation resulted in lower rates of in‐hospital and 30‐day mortality, hospitalisation stay and COVID‐19 disease progression; however, this positive effect was only seen among those with moderate COVID‐19 at baseline and not those with mild COVID‐19, suggesting that ACEIs/ARBs should be continued in patients with moderate COVID‐19 disease severity, especially as ACEIs/ARBs are well known to have substantial benefits for patients with hypertension and heart failure and thus stopping them would be deleterious; it is worth noting that because about 80% of patients were on ARBs, the observed benefits might not be extended to ACEIs.

### 4.1 Strengths and limitations

This review presents the most comprehensive systematic overview on the impact of RAAS inhibitors on COVID‐19‐related clinical outcomes, with a wide range of sensitivity (subgroup) analyses to assess the robustness of the evidence. None of the pooled meta‐analysis estimates for the nine studied outcomes was affected/dominated by an individual study. Although most of the included studies were classified as ‘low’ or ‘critically low’ quality using the AMSTAR 2 tool, it is widely acknowledged that the AMSTAR 2 tool has a high standard with most reviews rated as ‘critically low’.^74^, ^75^ The AMSTAR 2 tool is also prone to subjective biases,^76^ and assessment results are at the discretion of the reviewers regarding what is a ‘comprehensive’ literature search or ‘satisfactory’ explanation of heterogeneity or risk of bias assessment^76^; therefore, quality assessment was conducted fully independently in this review. Alternative tools to AMSTAR 2 exist, such as the ROBIS tool; however, the measurement categories are found to be broadly similar to the AMSTAR 2 tool which considered more reliable.^76^ An assessment of the degree of overlap among the 47 included reviews revealed a moderate degree of overlap of original studies with a calculated CCA of 9.2, with the CCA typically ranging from 1.5–11.4 for umbrella reviews (13). However, as three reviews did not provide full details of the included studies, this may be a slight underestimation. The overlap of primary studies within umbrella reviews is a well‐established limitation of this type of review and meta‐analysis,^77^ and we wish to stress to readers that this umbrella review provides a summary overview to offer insight into the current landscape of evidence (77). The moderate overlap identified highlights the unnecessary duplication of reviews conducted within this topic in 2020, which strongly relates to our observation that most of the eligible reviews did not prospectively register their protocol. Furthermore, this degree of overlap should be interpreted with caution because although a primary study might have been included in more than one review for different exposure–outcome relationships (hence, technically seen as an overlap), it might be included to assess different outcomes. It is plausible that data on different outcomes of a primary study could be extracted and included in two reviews. This is of particular relevance because many COVID‐19‐related clinical outcomes have been assessed (e.g. mortality, severity and hospitalisation) and not all the eligible reviews assessed the same outcomes. Therefore, a primary study might have been included in several reviews for different outcomes with no real overlap in data but misleadingly declared as an overlap (13). We have retained all the overlapping reviews as recommended by Okoth et al^78^ who suggested to retain the overlapping reviews when there is slight to moderate degree of overlap (CCA ≤10). Moreover, we do acknowledge that we might have missed potential review(s) that have been published after our search date, but we do believe that our umbrella review is the first study to comprehensively summarise the available evidence on the impact of ACEIs/ARBs on COVID‐19 clinical outcomes.

## 5 CONCLUSION

Collective evidence so far from observational studies indicates a good quality evidence on the significant association between ACEIs/ARBs use and reduction in death and death/ICU admission (as a composite outcome). Additionally, ACEIs/ARBs use was found to be associated with a significant reduction in severe COVID‐19 but a significant increase in hospitalisation; however, the evidence for these two outcomes was of poor quality; hence, cautious interpretation of these findings is required. Interestingly, findings for some of the clinical outcomes were dependent on whether the included patients had hypertension or not. Overall, our study findings further support the current recommendations of not discontinuing ACEIs/ARBs therapy in patients with COVID‐19 due to the lack of good quality evidence on their harm but rather it could be beneficial to patients.

## AUTHOR CONTRIBUTORS

NW and TM involved in data collection and management; AK involved in data analysis and interpretation; all authors involved in study conception and design, manuscript writing and drafting, manuscript reviewing and revising as well as providing constrictive criticism and final approval.

## FUNDING INFORMATION

None.

## CONFLICT OF INTEREST

Nothing to declare.

## Supporting information


Supplementary file S1

Supplementary file S2

Supplementary file S3

Supplementary file S4

Supplementary file S5

Supplementary file S6

Supplementary file S6A

Supplementary file S6B

Supplementary file S7

Supplementary file S7A

Supplementary file S7B

Supplementary file S8

Supplementary file S8A

Supplementary file S8B

Supplementary file S9

Supplementary file S9A

Supplementary file S9B

Supplementary file S10

Supplementary file S11
