# Supplementary material for: An umbrella review and meta‐analysis of renin–angiotensin system drugs use and COVID‐19 outcomes
Source: Eur J Clin Invest. 2022 Oct 19;53(2):e13888. doi: 10.1111/eci.13888 (PMC9874890; doi:10.1111/eci.13888)
Supplement: Supplementary file 1 — Supplementary file S1 [file ECI-53-0-s003.docx]

Supplementary File X: Search strategy

| **Database** | **Search terms** | **Results** |
| --- | --- | --- |
| **Medline** | (renin-angiotensin system.mp. OR Renin-Angiotensin System/ OR Angiotensin Receptor Antagonists/ OR Angiotensin II receptor Antagonists.mp. OR Angiotensin-Converting Enzyme Inhibitors/ OR ACEI OR ARB) AND (Coronavirus/ OR Coronavirus Infections/ OR COVID19.mp. OR COVID-19.mp. OR corona-virus.mp. OR severe acute respiratory syndrome coronavirus 2.mp.) AND Systematic Review/ OR systematic review.mp. OR meta-analys* OR Meta-Analysis/  Limit to English language and yr = 2019 – 2022 | 53 |
| **Embase** | (renin-angiotensin system.mp. OR renin angiotensin aldosterone system/ OR Angiotensin Receptor Antagonist/ OR Angiotensin converting Enzyme Inhibitors.mp. OR ACEI OR ARB) AND (Coronavirae/ OR coronavirus.mp. OR COVID-19.mp. OR COVID19.mp. OR severe acute respiratory syndrome coronavirus 2.mp.) AND systematic review/ OR systematic review.mp. OR meta analysis/ OR meta-analys*  Limit to English language and yr = 2019 – 2022 | 108 |
| **Scopus** | ( ( TITLE-ABS-KEY ( coronavirus ) ) OR ( TITLE-ABS-KEY ( covid19 ) ) OR ( TITLE-ABS-KEY ( corona-virus ) ) OR ( TITLE-ABS-KEY ( covid-19 ) ) OR ( TITLE-ABS-KEY ( "severe acute respiratory syndrome coronavirus 2" ) ) ) AND ( ( TITLE-ABS-KEY ( "ACEI" ) ) OR ( TITLE-ABS-KEY ( "ARB" ) ) OR ( TITLE-ABS-KEY ( "renin-angiotensin system" ) ) OR ( TITLE-ABS-KEY ( "Angiotensin Receptor Antagonists" ) ) OR ( TITLE-ABS-KEY ( "Angiotensin II Receptor Antagonists" ) ) OR ( TITLE-ABS-KEY ( "Angiotensin Converting Enzyme Inhibitors" ) ) ) AND ( ( TITLE-ABS-KEY ( "systematic review" ) ) OR ( TITLE-ABS-KEY ( meta-analys* ) ) ) AND ( LIMIT-TO ( LANGUAGE , "English" ) ) | 74 |
| **MedRixiv** | ("Systematic review" OR "meta-analysis" OR “meta-analyses”) AND ("renin-angiotensin system" OR "Angiotensin Receptor Antagonists" OR "Angiotensin II Receptor Antagonists" OR ACEI OR ARB OR "Angiotensin Converting Enzyme Inhibitors")  Accessed via NIH COVID-19 Portfolio (a database specific to COVID-19 hence no search terms for this)  Limit to search within title / abstract | 21 |
| **Cochrane Library** | (Renin-angiotensin system OR angiotensin receptor antagonists OR angiotensin 2 receptor antagonists OR angio-tensin converting enzyme inhibitors OR ARB OR ACEI) AND (coronavirus OR coronavirus infection OR COVID19 OR COVID-19 OR corona-virus OR severe acute respiratory syndrome coronavirus 2)  Search in ‘Cochrane Reviews’ | 0 |
