# Supplementary material for: An umbrella review and meta‐analysis of renin–angiotensin system drugs use and COVID‐19 outcomes
Source: Eur J Clin Invest. 2022 Oct 19;53(2):e13888. doi: 10.1111/eci.13888 (PMC9874890; doi:10.1111/eci.13888)
Supplement: Supplementary file 2 — Supplementary file S2 [file ECI-53-0-s006.docx]

**Irrelevant and excluded articles**

Article deems irrelevant at abstract/title stage (n=84) **(1-84)**

Articles excluded at full text review (n=26) (85-110)

- Duplicate review* (n=17) (85, 87-89, 91, 92, 95, 97, 98, 100-103, 105-107, 109)
- No meta-analysis conducted (n=5) (90, 93, 94, 99, 110)
- Abstract / conference proceeding (n=2) (104, 108)
- No results available (n=1) (86)
- Insufficient information available for meta-analysis (n=1) (96)

** For example, identified as a pre-print of a published review*

**References**

1. Acharya KP, Poudel P, Sherpa K, Adhikari CM. COVID-19: Cardiovascular perspective. Nepalese Heart Journal. 2020;17(1):1-5.

2. Ahmed S, Zimba O, Gasparyan AY. Thrombosis in Coronavirus disease 2019 (COVID-19) through the prism of Virchow's triad. Clinical Rheumatology. 2020;39(9):2529-43.

3. Akshayaa L, Girija S, Ezhilarasan AS. Association of ace expression among hypertension patients in Covid - 19 pandemic. European Journal of Molecular and Clinical Medicine. 2020;7(1):186-93.

4. Alkhatrya MS, Al Qadi SH, Dakhlala F, Zaiter IJ. COVID-19 pandemic and use of angiotensin-converting enzyme inhibitors (ACEI) and non-steroidal anti-inflammatory drugs (NSAIDs). American Journal of Gastroenterology. 2020;115 (SUPPL):S1719.

5. Alsaied T. From Other Journals: A Review of Recent Articles in Pediatric Cardiology. Pediatric cardiology. 2020;41(7):1532-7.

6. Baba R, Oki K, Itcho K, Kobuke K, Nagano G, Ohno H, et al. Angiotensin-converting enzyme 2 expression is not induced by the renin-angiotensin system in the lung. ERJ Open Research. 2020;6(4):1-3.

7. Bean D, Kraljevic Z, Searle T, Bendayan R, Pickles A, Folarin A, et al. ACE-inhibitors and Angiotensin-2 Receptor Blockers are not associated with severe SARS- COVID19 infection in a multi-site UK acute Hospital Trust2020.

8. Bean DM, Kraljevic Z, Searle T, Bendayan R, Kevin O, Pickles A, et al. Angiotensin-converting enzyme inhibitors and angiotensin II receptor blockers are not associated with severe COVID-19 infection in a multi-site UK acute hospital trust. European Journal of Heart Failure. 2020;22(6):967-74.

9. Chaudhary AP, Adna Nelson K, Jamuna TR. Smoking and covid-19: Renin-angiotensin system the hidden link. International Journal of Research in Pharmaceutical Sciences. 2020;11(4 Special issue 1):957-62.

10. Chen C, Wang F, Chen P, Jiang J, Cui G, Zhou N, et al. Mortality and Pre-Hospitalization use of Renin-Angiotensin System Inhibitors in Hypertensive COVID-19 Patients. Journal of the American Heart Association. 2020;9(21):e017736.

11. Coetzee A, Taljaard JJ, Hugo SS, Conradie M, Conradie-Smit M, Dave JA. Diabetes mellitus and COVID-19: A review and management guidance for South Africa. South African Medical Journal. 2020;110(8):761-6.

12. Cota LB, Pedrosa AL, de Brito SBCS, Froes ACF, de Carvalho ST, Fonseca GG, et al. COVID-19 and Renal Diseases: An Update. Current drug targets. 2020;13.

13. Dambha Miller H, Albasri A, Hodgson S, Wilcox CR, Khan S, Islam NN, et al. Currently prescribed drugs in the UK that could up or downregulate ACE2 in COVID-19 disease: A systematic review2020.

14. Dambha-Miller H, Albasri A, Hodgson S, Wilcox CR, Khan S, Islam N, et al. Currently prescribed drugs in the UK that could upregulate or downregulate ACE2 in COVID-19 disease: a systematic review. BMJ Open. 2020;10(9):e040644.

15. Dambha-Miller H, Albasri A, Hodgson S, Wilcox CR, Khan S, Islam N, et al. Currently prescribed drugs in the UK that could upregulate or downregulate ACE2 in COVID-19 disease: A systematic review. BMJ Open. 2020;10 (9) (no pagination)(e040644).

16. de Barcelos Ubaldo Martins L, Jabour L, Vieira CC, Nery LCC, Dias RF, Simoes ESAC. Renin-angiotensin system (RAS) and immune system profile in specific subgroups with COVID-19. Current Medicinal Chemistry. 2020;03:03.

17. Di Gennaro F, Pizzol D, Marotta C, Antunes M, Racalbuto V, Veronese N, et al. Coronavirus diseases (COVID-19) current status and future perspectives: A narrative review. International Journal of Environmental Research and Public Health. 2020;17(8).

18. Di Gennaro F, Pizzol D, Marotta C, Antunes M, Racalbuto V, Veronese N, et al. Coronavirus diseases (COVID-19) current status and future perspectives: A narrative review. International Journal of Environmental Research and Public Health. 2020;17 (8) (no pagination)(2690).

19. Dou Q, Wei X, Zhou K, Yang S, Jia P. Cardiovascular Manifestations and Mechanisms in Patients with COVID-19. Trends in Endocrinology & Metabolism. 2020;31(12):893-904.

20. Fedson DS. Statin Treatment of COVID-19. American Journal of Cardiology. 2020;136:171-3.

21. Fiesco-Sepúlveda KY, Serrano-Bermúdez LM. Contributions of latin American researchers in the understanding of the novel coronavirus outbreak: A literature review. PeerJ. 2020;8.

22. Fiesco-Sepulveda KY, Serrano-Bermudez LM. Contributions of latin American researchers in the understanding of the novel coronavirus outbreak: A literature review. PeerJ. 2020;8 (no pagination)(9332).

23. Gao C, Cai Y, Zhang K, Zhou L, Zhang Y, Zhang X, et al. Association of hypertension and antihypertensive treatment with COVID-19 mortality: a retrospective observational study. European Heart Journal. 2020;41(22):2058-66.

24. Gkogkou E, Barnasas G, Vougas K, Trougakos IP. Expression profiling meta-analysis of ACE2 and TMPRSS2, the putative anti-inflammatory receptor and priming protease of SARS-CoV-2 in human cells, and identification of putative modulators. Redox Biology. 2020;36:101615.

25. Gobolos L, Racz I, Hogan M, Remsey-Semmelweis E, Atallah B, AlMahmeed W, et al. The role of renin-angiotensin system activated phagocytes in the SARS-CoV-2 coronavirus infection. Journal of vascular surgery. 2020;18.

26. Gupta R, Misra A. Contentious issues and evolving concepts in the clinical presentation and management of patients with COVID-19 infectionwith reference to use of therapeutic and other drugs used in Co-morbid diseases (Hypertension, diabetes etc). Diabetes and Metabolic Syndrome: Clinical Research and Reviews. 2020;14(3):251-4.

27. Hatami N, Ahi S, Sadeghinikoo A, Foroughian M, Javdani F, Kalani N, et al. Worldwide ACE (I/D) polymorphism may affect COVID-19 recovery rate: an ecological meta-regression. Endocrine. 2020;68(3):479-84.

28. Ho JSY, Tambyah PA, Ho AFW, Chan MYY, Sia CH. Effect of coronavirus infection on the human heart: A scoping review. European Journal of Preventive Cardiology. 2020;27(11):1136-48.

29. Hu T, Liu Y, Zhao M, Zhuang Q, Xu L, He Q. A comparison of COVID-19, SARS and MERS. PeerJ. 2020;8 (no pagination)(e9725).

30. Huang I, Lim MA, Pranata R. Diabetes mellitus is associated with increased mortality and severity of disease in COVID-19 pneumonia - A systematic review, meta-analysis, and meta-regression: Diabetes and COVID-19. Diabetes and Metabolic Syndrome: Clinical Research and Reviews. 2020;14(4):395-403.

31. Hussain A, Bhowmik B, do Vale Moreira NC. COVID-19 and diabetes: Knowledge in progress. Diabetes Research and Clinical Practice. 2020;162.

32. Hussain A, Bhowmik B, do Vale Moreira NC. COVID-19 and diabetes: Knowledge in progress. Diabetes Research and Clinical Practice. 2020;162 (no pagination)(108142).

33. Jin Y, Ji W, Yang H, Chen S, Zhang W, Duan G. Endothelial activation and dysfunction in COVID-19: from basic mechanisms to potential therapeutic approaches. Signal Transduction and Targeted Therapy. 2020;5(1).

34. Kaur U, Acharya K, Mondal R, Singh A, Saso L, Chakrabarti S, et al. Should ACE2 be given a chance in COVID-19 therapeutics: A semi-systematic review of strategies enhancing ACE2. European Journal of Pharmacology. 2020;887.

35. Kaur U, Acharya K, Mondal R, Singh A, Saso L, Chakrabarti S, et al. Should ACE2 be given a chance in COVID-19 therapeutics: A semi-systematic review of strategies enhancing ACE2. European Journal of Pharmacology. 2020;887 (no pagination)(173545).

36. Kolin DA, Kulm S, Christos PJ, Elemento O. Clinical, regional, and genetic characteristics of Covid-19 patients from UK Biobank. PLoS ONE. 2020;15(11 November).

37. Kolin DA, Kulm S, Christos PJ, Elemento O. Clinical, regional, and genetic characteristics of Covid-19 patients from UK Biobank. PLoS ONE. 2020;15 (11 November) (no pagination)(e0241264).

38. Kow CS, Hasan SS. Do the meta-analyses provide a clean bill of health to the use of renin-angiotensin system inhibitors in COVID-19? Clinical infectious diseases : an official publication of the Infectious Diseases Society of America. 2020;08.

39. Liu Y, Huang F, Xu J, Yang P, Qin Y, Cao M, et al. Anti-hypertensive Angiotensin II receptor blockers associated to mitigation of disease severity in elderly COVID-19 patients2020.

40. Maglakelidze N, Manto KM, Craig TJ. A Review: Does Complement or the Contact System Have a Role in Protection or Pathogenesis of COVID-19? Pulmonary Therapy. 2020;6(2):169-76.

41. Maldonado V, Loza-Mejía MA, Chávez-Alderete J. Repositioning of pentoxifylline as an immunomodulator and regulator of the renin-angiotensin system in the treatment of COVID-19. Medical Hypotheses. 2020;144.

42. Maldonado V, Loza-Mejia MA, Chavez-Alderete J. Repositioning of pentoxifylline as an immunomodulator and regulator of the renin-angiotensin system in the treatment of COVID-19. Medical Hypotheses. 2020;144 (no pagination)(109988).

43. Marhl M, Grubelnik V, Magdič M, Markovič R. Diabetes and metabolic syndrome as risk factors for COVID-19. Diabetes and Metabolic Syndrome: Clinical Research and Reviews. 2020;14(4):671-7.

44. Matsushita K, Ding N, Kou M, Hu X, Chen M, Gao Y, et al. The relationship of COVID-19 severity with cardiovascular disease and its traditional risk factors: A systematic review and meta-analysis2020.

45. Matsushita K, Ding N, Kou M, Hu X, Chen M, Gao Y, et al. The relationship of COVID-19 severity with cardiovascular disease and its traditional risk factors: A systematic review and meta-analysis. Global Heart. 2020;15 (1) (no pagination)(64).

46. Mehta N, Mazer-Amirshahi M, Alkindi N, Pourmand A. Pharmacotherapy in COVID-19; A narrative review for emergency providers. American Journal of Emergency Medicine. 2020;38(7):1488-93.

47. Michaud V, Deodhar M, Arwood M, Al Rihani SB, Dow P, Turgeon J. ACE2 as a therapeutic target for COVID-19; its role in infectious processes and regulation by modulators of the raas system. Journal of Clinical Medicine. 2020;9(7):1-27.

48. Miners S, Kehoe PG, Love S. Cognitive impact of COVID-19: looking beyond the short term. Alzheimer's Research and Therapy. 2020;12(1).

49. Miners S, Kehoe PG, Love S. Cognitive impact of COVID-19: looking beyond the short term. Alzheimer's Research and Therapy. 2020;12 (1) (no pagination)(170).

50. Mitchell F. Vitamin-D and COVID-19: do deficient risk a poorer outcome? The Lancet Diabetes and Endocrinology. 2020;8(7):570.

51. Morales DR, Conover MM, You SC, Pratt N, Kostka K, Duarte-Salles T, et al. Renin-angiotensin system blockers and susceptibility to COVID-19: an international, open science, cohort analysis. The Lancet Digital Health. 2021;3(2):e98-e114.

52. Moran SM, Barbour S, Dipchand C, Garland JS, Hladunewich M, Jauhal A, et al. Management of Patients With Glomerulonephritis During the COVID-19 Pandemic: Recommendations From the Canadian Society of Nephrology COVID-19 Rapid Response Team. Canadian Journal of Kidney Health and Disease. 2020;7.

53. Muchtaridi M, Fauzi M, Ikram NKK, Gazzali AM, Wahab HA. Natural Flavonoids as Potential Angiotensin-Converting Enzyme 2 Inhibitors for Anti-SARS-CoV-2. Molecules. 2020;25(17).

54. Ortiz-Prado E, Simbaña-Rivera K, Gómez- Barreno L, Rubio-Neira M, Guaman LP, Kyriakidis NC, et al. Clinical, molecular, and epidemiological characterization of the SARS-CoV-2 virus and the Coronavirus Disease 2019 (COVID-19), a comprehensive literature review. Diagnostic Microbiology and Infectious Disease. 2020;98(1).

55. Ortiz-Prado E, Simbana-Rivera K, Gomez- Barreno L, Rubio-Neira M, Guaman LP, Kyriakidis NC, et al. Clinical, molecular, and epidemiological characterization of the SARS-CoV-2 virus and the Coronavirus Disease 2019 (COVID-19), a comprehensive literature review. Diagnostic Microbiology and Infectious Disease. 2020;98 (1) (no pagination)(115094).

56. Oz M, Lorke DE, Kabbani N. A comprehensive guide to the pharmacologic regulation of angiotensin converting enzyme 2 (ACE2), the SARS-CoV-2 entry receptor. Pharmacology and Therapeutics. 2021;221 (no pagination)(107750).

57. Panarese A, Shahini E. Letter: Covid-19, and vitamin D. Alimentary Pharmacology and Therapeutics. 2020;51(10):993-5.

58. Pezeshki S, Parvaresh S, Sinaei R. Why COVID-19 is less frequent and severe in children: a narrative review. World Journal of Pediatrics. 2020.

59. Porter GJ. Will children reveal their secret? The coronavirus dilemma. European Respiratory Journal. 2020;55(6).

60. Porter GJ. Will children reveal their secret? The coronavirus dilemma. European Respiratory Journal. 2020;55 (6) (no pagination)(2001382).

61. Russo V, Bottino R, Carbone A, Rago A, Papa AA, Golino P, et al. Covid-19 and heart: From clinical features to pharmacological implications. Journal of Clinical Medicine. 2020;9(6):1-19.

62. Samidurai A, Das A. Cardiovascular complications associated with COVID-19 and potential therapeutic strategies. International Journal of Molecular Sciences. 2020;21(18):1-28.

63. Sanders JM, Monogue ML, Jodlowski TZ, Cutrell JB. Pharmacologic Treatments for Coronavirus Disease 2019 (COVID-19): A Review. JAMA - Journal of the American Medical Association. 2020;323(18):1824-36.

64. Sathish T, Cao Y. Is newly diagnosed diabetes as frequent as preexisting diabetes in COVID-19 patients? Diabetes and Metabolic Syndrome: Clinical Research and Reviews. 2021;15(1):147-8.

65. Schellack N, Coetzee M, Schellack G, Gijzelaar M, Hassim Z, Milne M, et al. COVID-19: Guidelines for pharmacists in South Africa. SA Pharmaceutical Journal. 2020;87(3):13-21.

66. Shankar M, Nishanth KR. SARS-coronavirus disease 19 and comorbidities- a systematic review. Journal of Clinical and Diagnostic Research. 2020;14(12):OE01-OE6.

67. Sidarta-Oliveira D, Jara CP, Ferruzzi AJ, Skaf MS, Velander WH, Araujo EP, et al. SARS-CoV-2 receptor is co-expressed with elements of the kinin-kallikrein, renin-angiotensin and coagulation systems in alveolar cells. Scientific reports. 2020;10(1):19522.

68. Singh SP, Sharma P, Singh D, Kumar P, Sharma R. Effect of coronavirus disease in patients with kidney disease in India. International Journal of Research in Pharmaceutical Sciences. 2020;11(Special Issue 1):1255-9.

69. Springate C, Martin A, Ghosh B, Gould H, Rice H, Rutherford L, et al. PRS52 RAPID Review of ACUTE Respiratory Distress Syndrome in Adults. Value in Health. 2020;23 (Supplement 2):S726.

70. Sugimoto T, Mizuno A, Kishi T, Ito N, Matsumoto C, Fukuda M, et al. Coronavirus disease 2019 (Covid-19) information for cardiologists - systematic literature review and additional analysis. Circulation Journal. 2020;84(6):1039-43.

71. Tajbakhsh A, Gheibi Hayat SM, Taghizadeh H, Akbari A, inabadi M, Savardashtaki A, et al. COVID-19 and cardiac injury: clinical manifestations, biomarkers, mechanisms, diagnosis, treatment, and follow up. Expert Review of Anti Infective Therapy. 2020.

72. Teoh CW, Gaudreault-Tremblay MM, Blydt-Hansen TD, Goldberg A, Arora S, Feber J, et al. Management of Pediatric Kidney Transplant Patients During the COVID-19 Pandemic: Guidance From the Canadian Society of Transplantation Pediatric Group. Canadian Journal of Kidney Health and Disease. 2020;7(no pagination).

73. Varshney AS, Wang DE, Bhatt AS, Blood A, Sharkawi MA, Siddiqi HK, et al. Characteristics of clinical trials evaluating cardiovascular therapies for Coronavirus Disease 2019 Registered on ClinicalTrials.gov: a cross sectional analysis. American Heart Journal. 2021;232:105-15.

74. Vazquez-Cornejo E. Considerations on the use of antihypertensive blockers of the renin-angiotensin system in adults and children in the face of the covid-19 pandemic. Boletin Medico del Hospital Infantil de Mexico. 2020;77(5):274-81.

75. Vieira C, Nery L, Martins L, Jabour L, Dias R, Simoes ESAC. Downregulation of Membrane-bound Angiotensin Converting Enzyme 2 (ACE2) Receptor has a Pivotal Role in COVID-19 Immunopathology. Current drug targets. 2020;20.

76. Vogel JP, Tendal B, Giles M, Whitehead C, Burton W, Chakraborty S, et al. Clinical care of pregnant and postpartum women with COVID-19: Living recommendations from the National COVID-19 Clinical Evidence Taskforce. Australian and New Zealand Journal of Obstetrics and Gynaecology. 2020;60(6):840-51.

77. Volpe M, Battistoni A. Systematic review of the role of renin-angiotensin system inhibitors in late studies on Covid-19: A new challenge overcome? International Journal of Cardiology. 2020;321:150-4.

78. Wang D, Li Z, Liu Y. An overview of the safety, clinical application and antiviral research of the COVID-19 therapeutics. Journal of Infection and Public Health. 2020;13(10):1405-14.

79. Wang Z, Wang D, Dai Y, Zhu S, Zeng H. Urogenital System Damaging Manifestations of Three Human Infected Coronaviruses. The Journal of urology. 2020:101097JU0000000000001400.

80. World Health Organization. COVID-19 and the use of angiotensin-converting enzyme inhibitors and receptor blockers. Scientific brief. Pediatria i Medycyna Rodzinna. 2020;16(1):120-1.

81. Xiang Y, Wong KCY, Hon Cheong SO. Exploring drugs and vaccines associated with altered risks and severity of COVID-19: a UK Biobank cohort study of all ATC level-4 drug categories2020.

82. Ye Q, Lu D, Shang S, Fu J, Gong F, Shu Q, et al. Crosstalk between coronavirus disease 2019 and cardiovascular disease and its treatment. ESC Heart Failure. 2020;7(6):3464-72.

83. Zhao J, Cui W, Tian BP. Efficacy of tocilizumab treatment in severely ill COVID-19 patients. Critical Care. 2020;24(1).

84. Zhao J, Cui W, Tian BP. Efficacy of tocilizumab treatment in severely ill COVID-19 patients. Critical Care. 2020;24 (1) (no pagination)(524).

85. Zhang X, Yu J, Pan LY, Jiang HY. ACEI/ARB use and risk of infection or severity or mortality of COVID-19: A systematic review and meta-analysis. Pharmacological Research. 2020;158 (no pagination)(104927).

86. Yang Y, Liu X, Liu Y, Chen Y, Tian Y, Fu X, et al. Efficacy and safety of ACEI/ARB drugs in patients with COVID-19 combined with diabetes mellitus: A protocol for systematic review and meta-analysis of randomized controlled trials. Medicine. 2020;99(35):e21723.

87. Usman MS, Siddiqi TJ, Khan MS, Ahmed A, Ali SS, Michos ED, et al. A Meta-analysis of the Relationship Between Renin-Angiotensin-Aldosterone System Inhibitors and COVID-19. The American journal of cardiology. 2020;02.

88. Ssentongo AE, Ssentongo P, Heilbrunn ES, Lekoubou A, Du P, Liao D, et al. Renin-angiotensin-aldosterone system inhibitors and the risk of mortality in patients with hypertension hospitalised for COVID-19: Systematic review and meta-analysis. Open Heart. 2020;7 (2) (no pagination)(e001353).

89. Ssentongo A, Ssentongo P, Heilbrunn ES, Lekoubou A, Du P, Liao D, et al. Renin-angiotensin-aldosterone system inhibitors and mortality in patients with hypertension hospitalized for COVID-19: a systematic review and meta-analysis2020.

90. Singh AK, Gupta R, Misra A. Comorbidities in COVID-19: Outcomes in hypertensive cohort and controversies with renin angiotensin system blockers. Diabetes and Metabolic Syndrome: Clinical Research and Reviews. 2020;14(4):283-7.

91. Patoulias D, Katsimardou A, Stavropoulos K, Imprialos K, Kalogirou MS, Doumas M. Renin-Angiotensin System Inhibitors and COVID-19: a Systematic Review and Meta-Analysis. Evidence for Significant Geographical Disparities. Current Hypertension Reports. 2020;22 (11) (no pagination)(90).

92. Mehraeen E, Karimi A, Barzegary A, Vahedi F, Afsahi AM, Dadras O, et al. Predictors of mortality in patients with COVID-19-a systematic review. European Journal of Integrative Medicine. 2020;40 (no pagination)(101226).

93. Mehraeen E, Karimi A, Barzegary A, Vahedi F, Afsahi AM, Dadras O, et al. Predictors of mortality in patients with COVID-19–a systematic review. European Journal of Integrative Medicine. 2020;40.

94. Mackey K, King VJ, Gurley S, Kiefer M, Liederbauer E, Vela K, et al. Risks and Impact of Angiotensin-Converting Enzyme Inhibitors or Angiotensin-Receptor Blockers on SARS-CoV-2 Infection in Adults: A Living Systematic Review. Annals of internal medicine. 2020;173(3):195-203.

95. Liu X, Long C, Xiong Q, Ma J, Chen C, Su Y, et al. Association of Renin-Angiotensin-Aldosterone System Inhibition with Risk of COVID-19, Inflammation Level Severity and Death in Patients With COVID-19: A Rapid Systematic Review and Meta-Analysis2020.

96. Lee KH, Kim JS, Hong SH, Seong D, Choi YR, Ahn YT, et al. Risk factors of COVID-19 mortality: a systematic review of current literature and lessons from recent retracted articles. Eur Rev Med Pharmacol Sci. 2020;24(24):13089-97.

97. Lee KH, Kim JS, Hong SH, Seong D, Choi YR, Ahn YT, et al. Risk factors of COVID-19 mortality: a systematic review of current literature and lessons from recent retracted articles. European Review for Medical & Pharmacological Sciences. 2020;24(24):13089-97.

98. Kurdi A, Abutheraa N, Akil L, Godman B. A systematic review and meta-analysis of the use of renin-angiotensin system drugs and COVID-19 clinical outcomes: What is the evidence so far? Pharmacology Research and Perspectives. 2020;8 (6) (no pagination)(e00666).

99. Freitas RFD, Torres SC, Nunes JPL. Syncope and COVID-19 disease – a systematic review2021.

100. Flacco ME, Acuti Martellucci C, Bravi F, Parruti G, Cappadona R, Mascitelli A, et al. Treatment with ACE inhibitors or ARBs and risk of severe/lethal COVID-19: a meta-analysis. Heart. 2020;01.

101. Di Castelnuovo A, Costanzo S, Antinori A, Berselli N, Blandi L, Bonaccio M, et al. RAAS inhibitors are not associated with mortality in COVID-19 patients: Findings from an observational multicenter study in Italy and a meta-analysis of 19 studies. Vascular Pharmacology. 2020;135.

102. De Almeida-Pititto B, Dualib PM, Zajdenverg L, Dantas JR, De Souza FD, Rodacki M, et al. Severity and mortality of COVID 19 in patients with diabetes, hypertension and cardiovascular disease: A meta-analysis. Diabetology and Metabolic Syndrome. 2020;12 (1) (no pagination)(75).

103. Covid RISK Treatments Collaboration. RAAS inhibitors are not associated with mortality in COVID-19 patients: Findings from an observational multicenter study in Italy and a meta-analysis of 19 studies. Vascular Pharmacology. 2020;135:106805.

104. Chu C, Zeng S, Hasan AA, Hocher CF, Kramer BK, Hocher B. Renin-angiotensin-aldosterone system blocking drugs in patients with SARS-COV-2: Systematic review and meta-analysis. Journal of the American Society of Nephrology. 2020;31:300.

105. Caldeira D, Alves M, Gouveia e Melo R, Silverio Antonio P, Cunha N, Nunes-Ferreira A, et al. Angiotensin-converting enzyme inhibitors and angiotensin-receptor blockers and the risk of COVID-19 infection or severe disease: Systematic review and meta-analysis. IJC Heart and Vasculature. 2020;31 (no pagination)(100627).

106. Baral R, White M, Vassiliou VS. Effect of Renin-Angiotensin-Aldosterone System Inhibitors in Patients with COVID-19: a Systematic Review and Meta-analysis of 28,872 Patients. Current Atherosclerosis Reports. 2020;22 (10) (no pagination)(61).

107. Baral R, White M, Vassiliou VS. Impact of hospitalised patients with COVID-19 taking Renin-Angiotensin-Aldosterone System inhibitors: a systematic review and meta-analysis2020.

108. Ali H, Mohamed MM, Daoud A, Fulop T, Posadas MAC, Casey M, et al. Raas inhibition, mortality, and severity in COVID-19 patients: A systematic review and meta-analysis. Journal of the American Society of Nephrology. 2020;31:299.

109. Alamer A, Abraham I. Mortality in COVID-19 patients treated with ACEIs/ARBs: Re-estimated meta-analysis results following the Mehra et al. retraction. Pharmacological Research. 2020;160.

110. Akhtar S, Benter IF, Danjuma MI, Doi SAR, Hasan SS, Habib AM. Pharmacotherapy in COVID-19 patients: a review of ACE2-raising drugs and their clinical safety. Journal of Drug Targeting. 2020;28(7-8):683-99.
