# Supplementary material for: An umbrella review and meta‐analysis of renin–angiotensin system drugs use and COVID‐19 outcomes
Source: Eur J Clin Invest. 2022 Oct 19;53(2):e13888. doi: 10.1111/eci.13888 (PMC9874890; doi:10.1111/eci.13888)
Supplement: Supplementary file 6 — Supplementary file S6 [file ECI-53-0-s014.pdf]

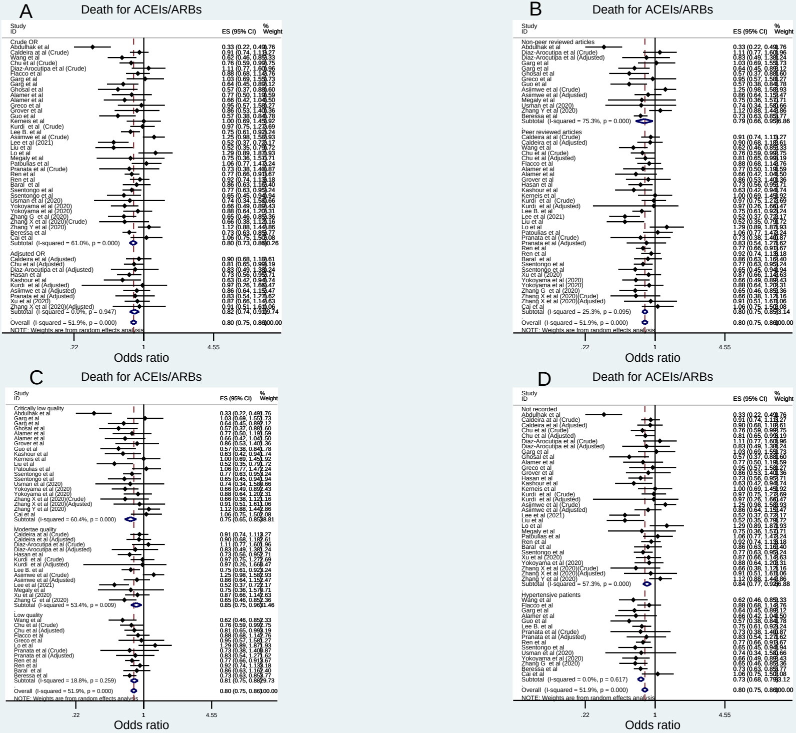
A
Death for ACEIs/ARBs
Study ID
ES (95% CI)
% Weight
Crude OR
Adjsuted OR
Overall (I-squared = 51.9%, p = 0.000)
NOTE: Weights are from random effects analysis
.22
1
4.55
Odds ratio
B
Death for ACEIs/ARBs
Non-peer reviewed articles
Peer reviewed articles
Overall (I-squared = 51.9%, p = 0.000)
NOTE: Weights are from random effects analysis
Odds ratio
C
Death for ACEIs/ARBs
Critically low quality
Modertae quality
Low quality
Overall (I-squared = 51.9%, p = 0.000)
NOTE: Weights are from random effects analysis
Odds ratio
D
Death for ACEIs/ARBs
Not recorded
Hypertensive patients
Overall (I-squared = 51.9%, p = 0.000)
NOTE: Weights are from random effects analysis
Odds ratio
