# Supplementary figures and images for: An umbrella review and meta‐analysis of renin–angiotensin system drugs use and COVID‐19 outcomes
Source: Eur J Clin Invest. 2022 Oct 19;53(2):e13888. doi: 10.1111/eci.13888 (PMC9874890; doi:10.1111/eci.13888)

A

Death for ACEIs

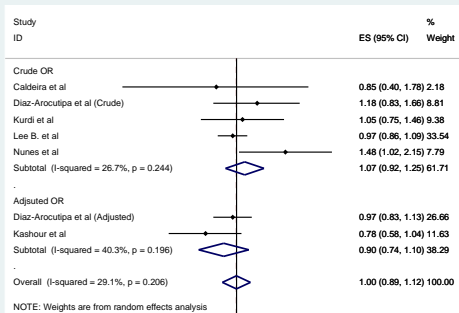

B

Death for ACEIs

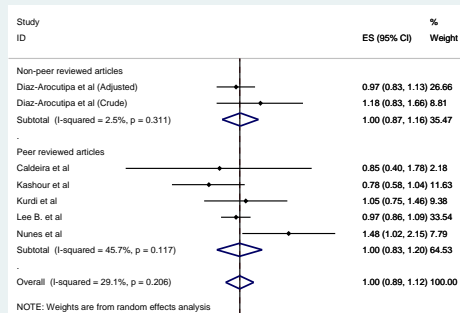

C

Death for ACEIs

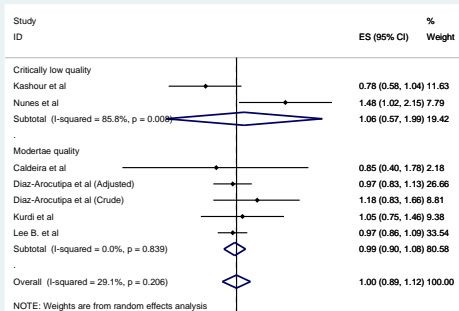

D

Death for ACEIs

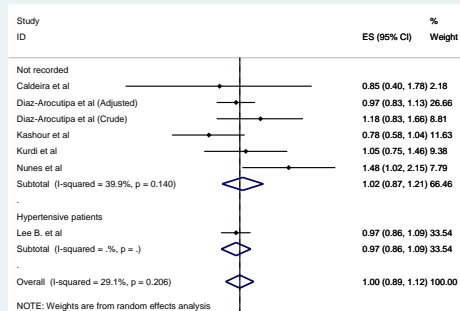

Supplement: Supplementary file 7 — Supplementary file S6A [file ECI-53-0-s007.pdf]

A

Death for ARBs

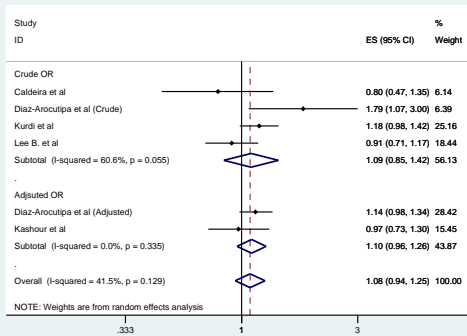

B

Death for ARBs

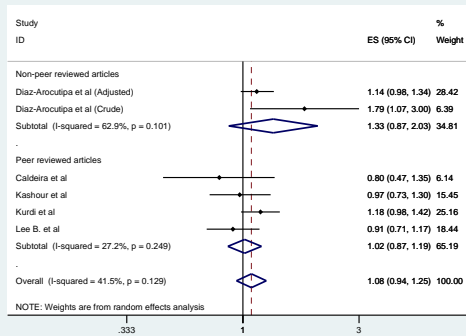

C

Death for ARBs

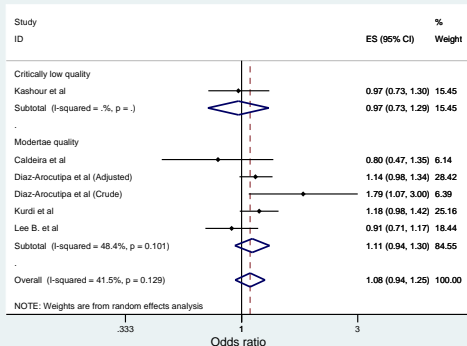

D

Death for ARBs

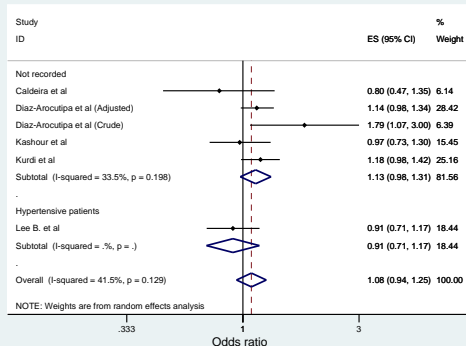

Supplement: Supplementary file 8 — Supplementary file S6B [file ECI-53-0-s017.pdf]

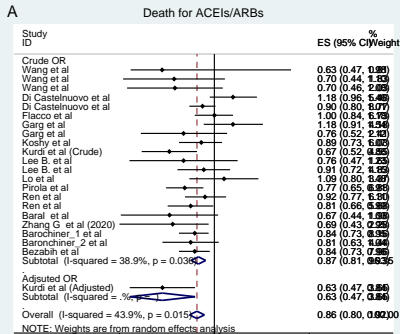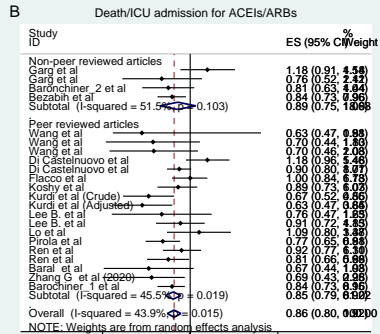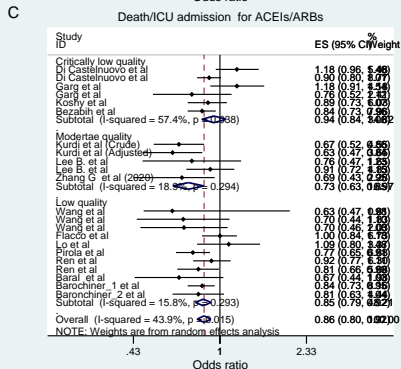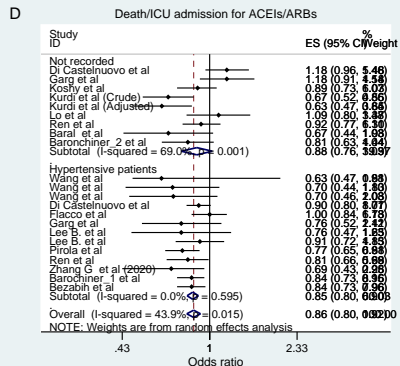

Supplement: Supplementary file 9 — Supplementary file S7 [file ECI-53-0-s008.pdf]

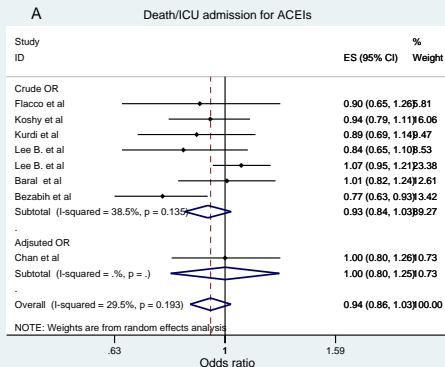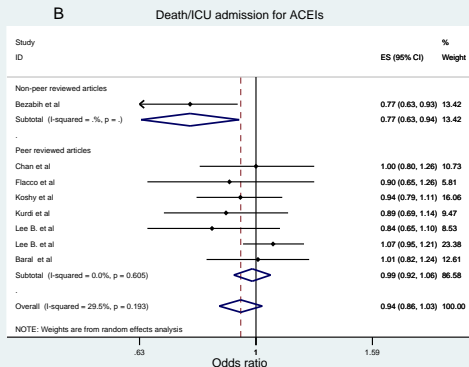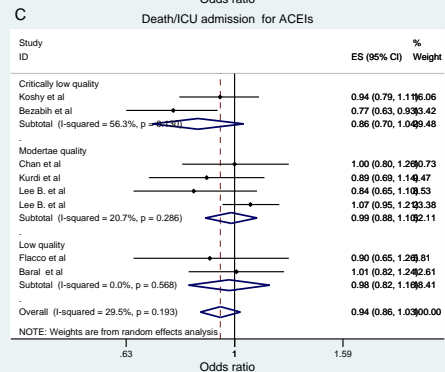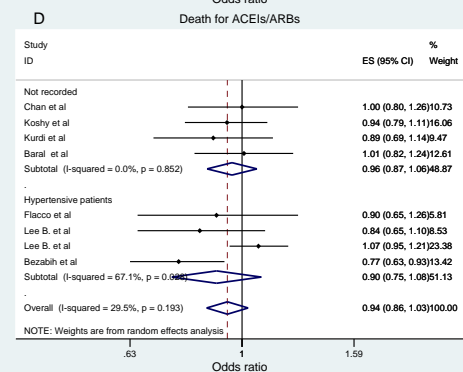

Supplement: Supplementary file 10 — Supplementary file S7A [file ECI-53-0-s019.pdf]

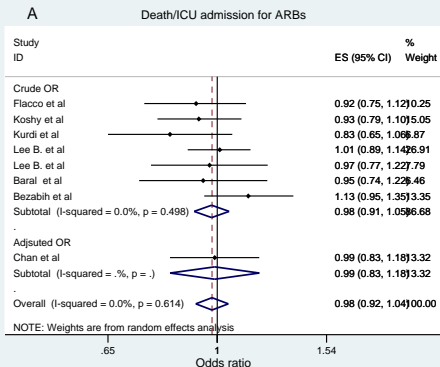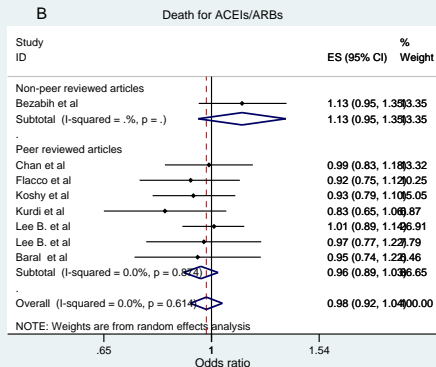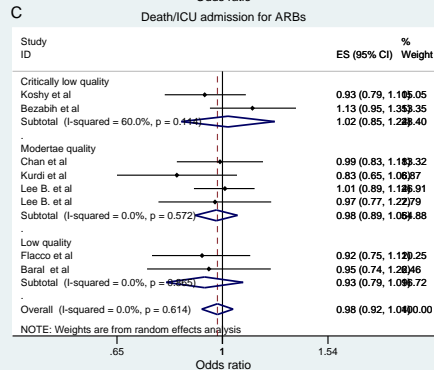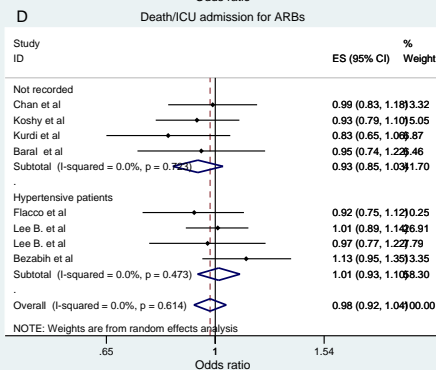

Supplement: Supplementary file 11 — Supplementary file S7B [file ECI-53-0-s015.pdf]

A

Severe COVID-19 for ARBs

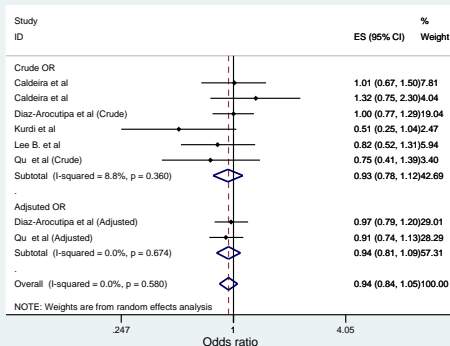

B

Severe COVID-19 for ARBs

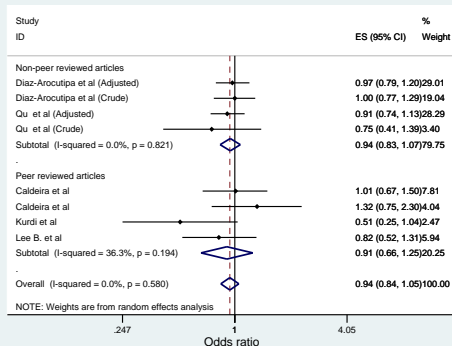

C

Severe COVID-19 for ARBs

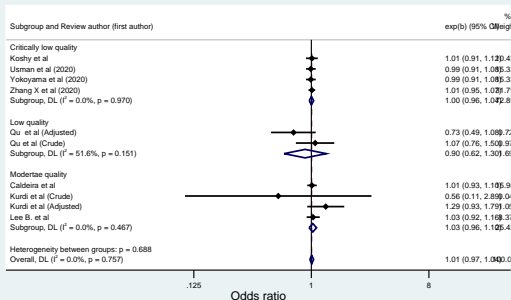

D

Severe COVID-19 for ARBs

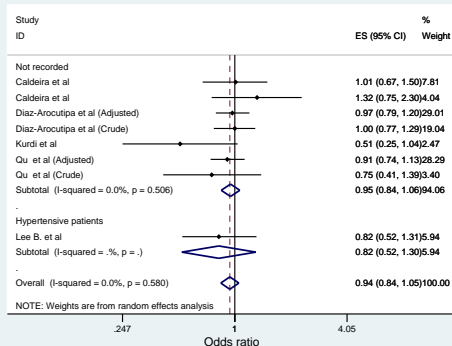

Supplement: Supplementary file 14 — Supplementary file S8B [file ECI-53-0-s011.pdf]

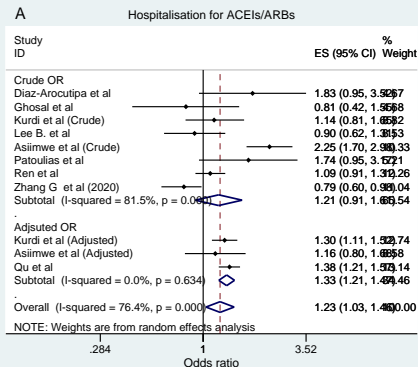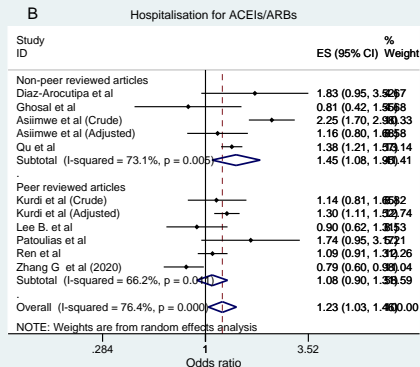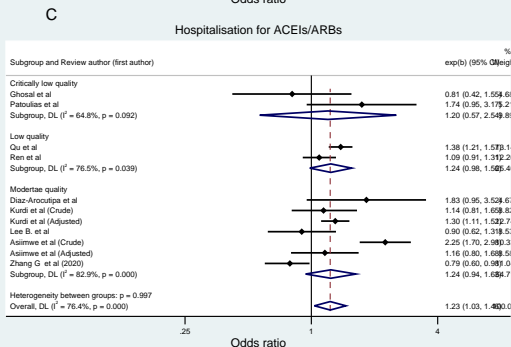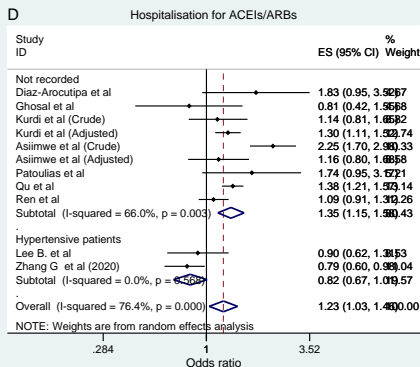

Supplement: Supplementary file 15 — Supplementary file S9 [file ECI-53-0-s001.pdf]

Supplementary file 10. Publication bias funnel plot for the outcomes with  $\geq 10$  studies

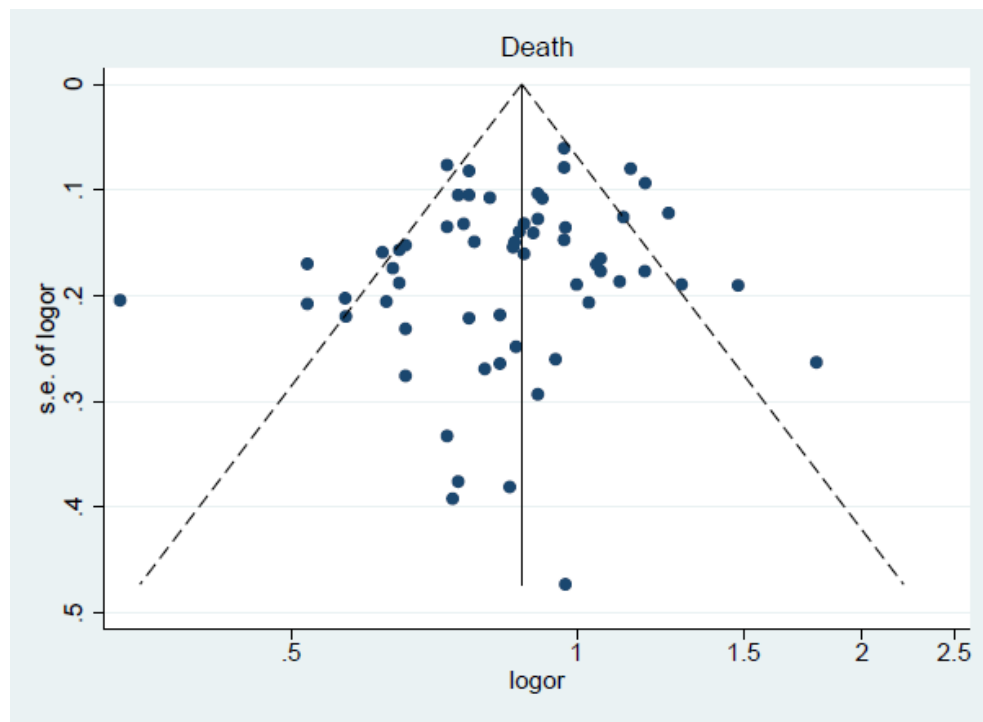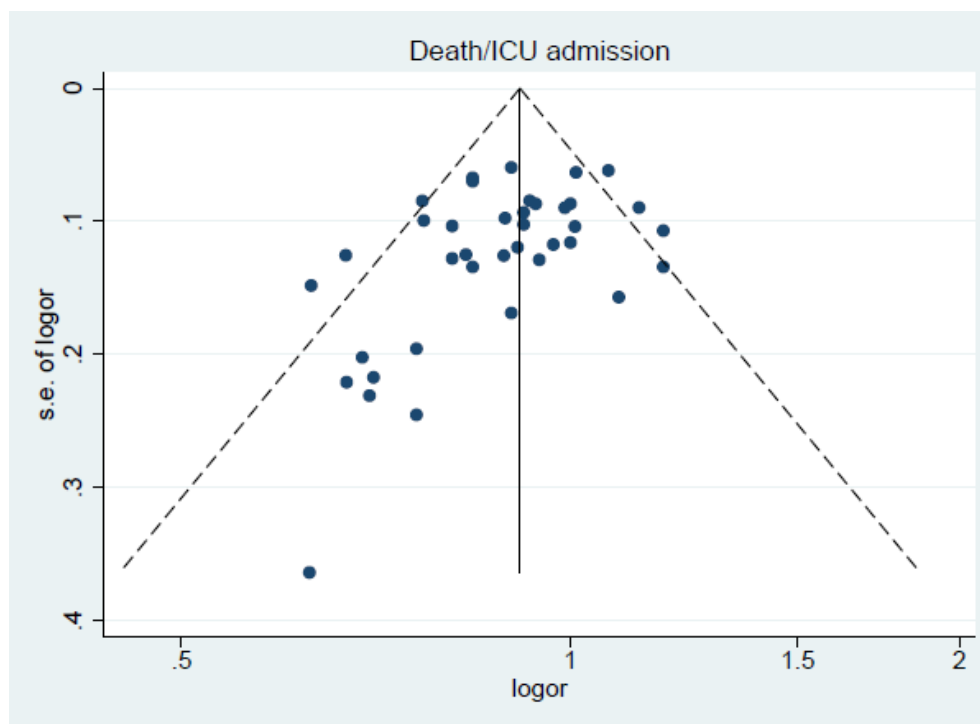

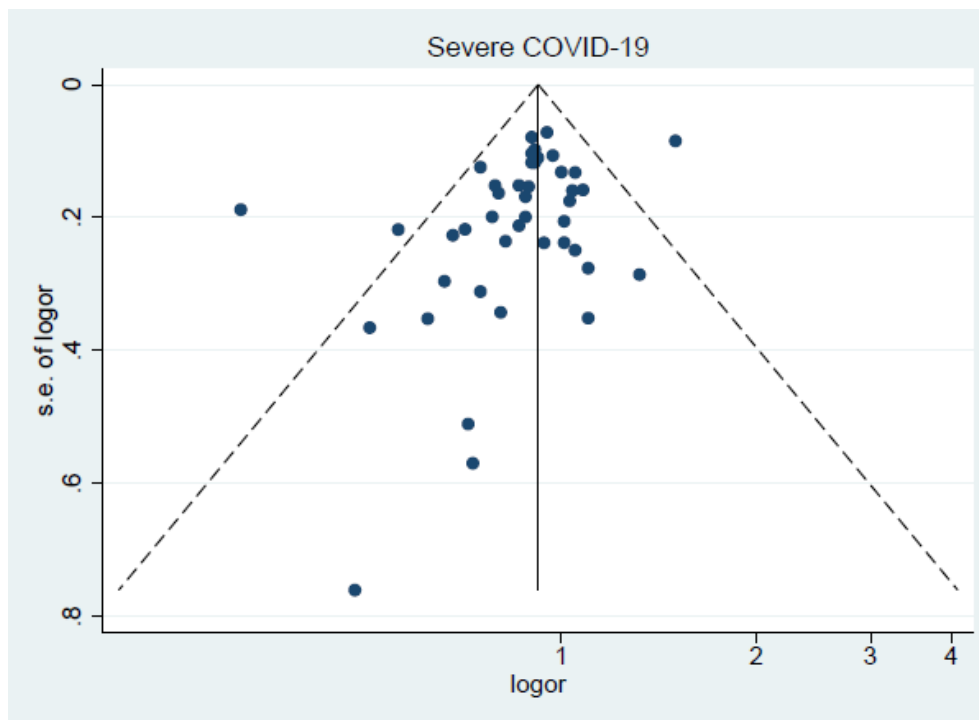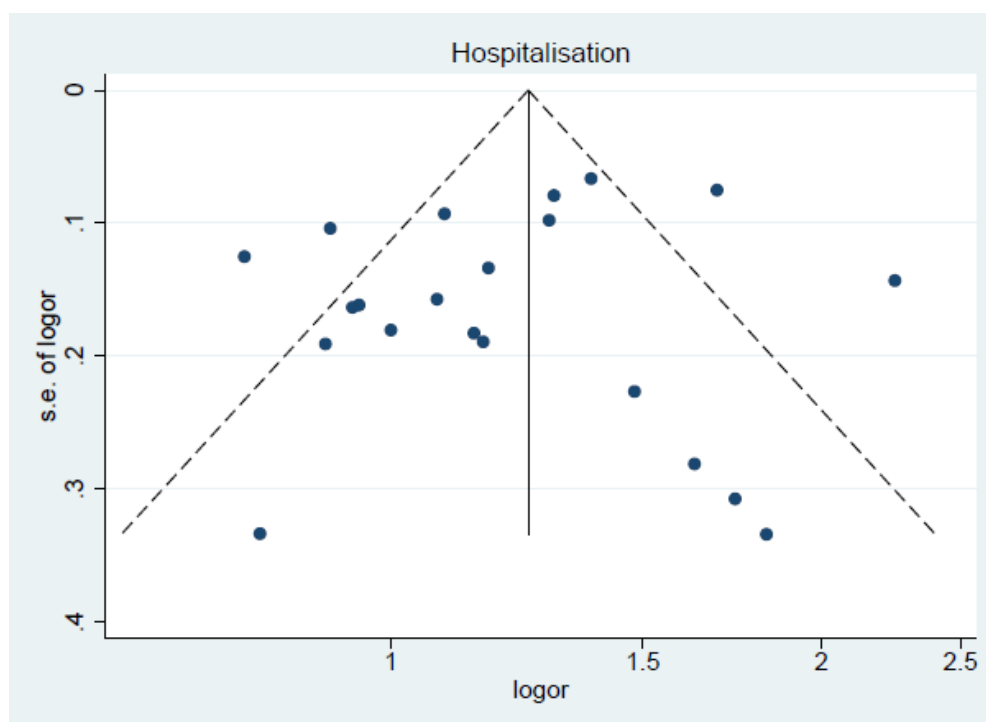

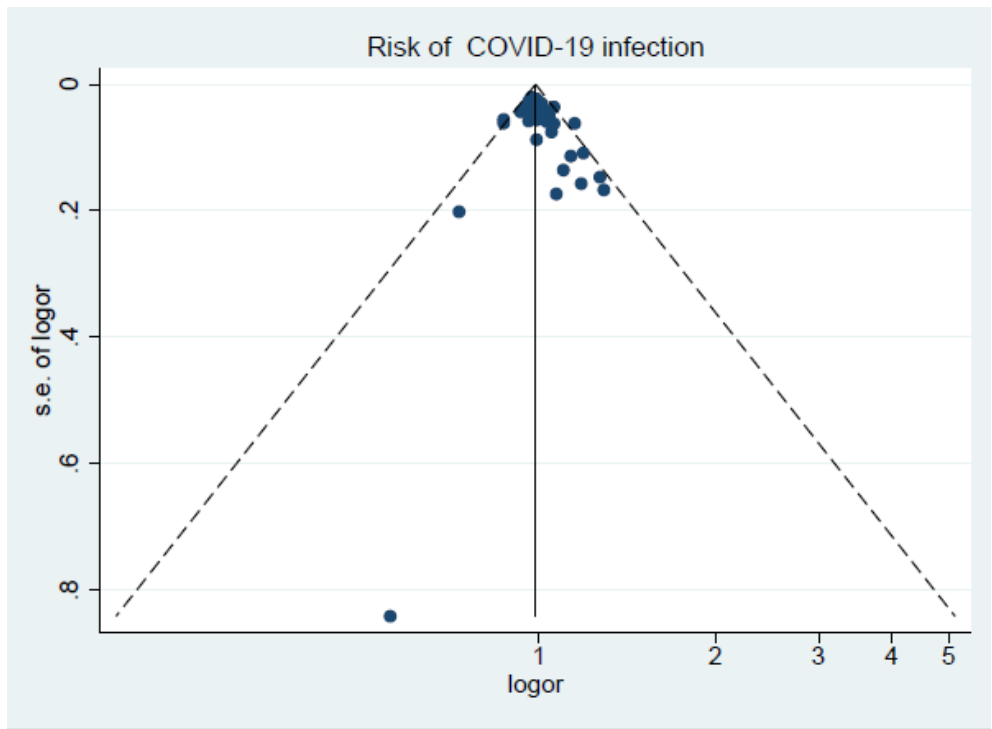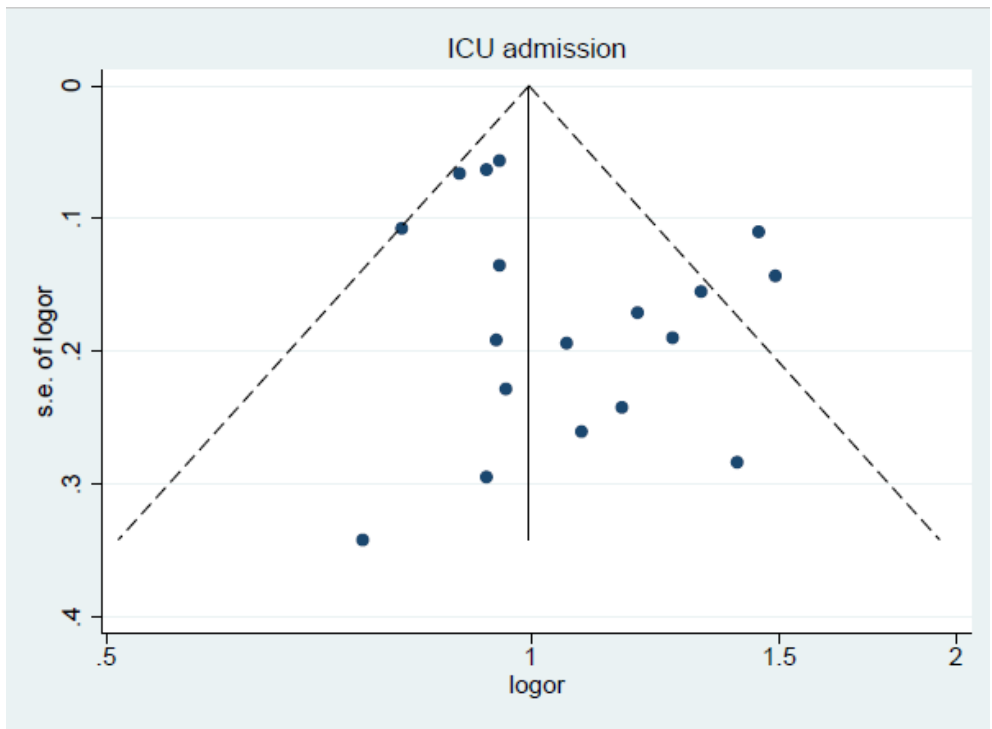

Supplement: Supplementary file 18 — Supplementary file S10 [file ECI-53-0-s004.pdf]
