# Supplementary material for: An umbrella review and meta‐analysis of renin–angiotensin system drugs use and COVID‐19 outcomes
Source: Eur J Clin Invest. 2022 Oct 19;53(2):e13888. doi: 10.1111/eci.13888 (PMC9874890; doi:10.1111/eci.13888)
Supplement: Supplementary file 12 — Supplementary file S8 [file ECI-53-0-s018.pdf]

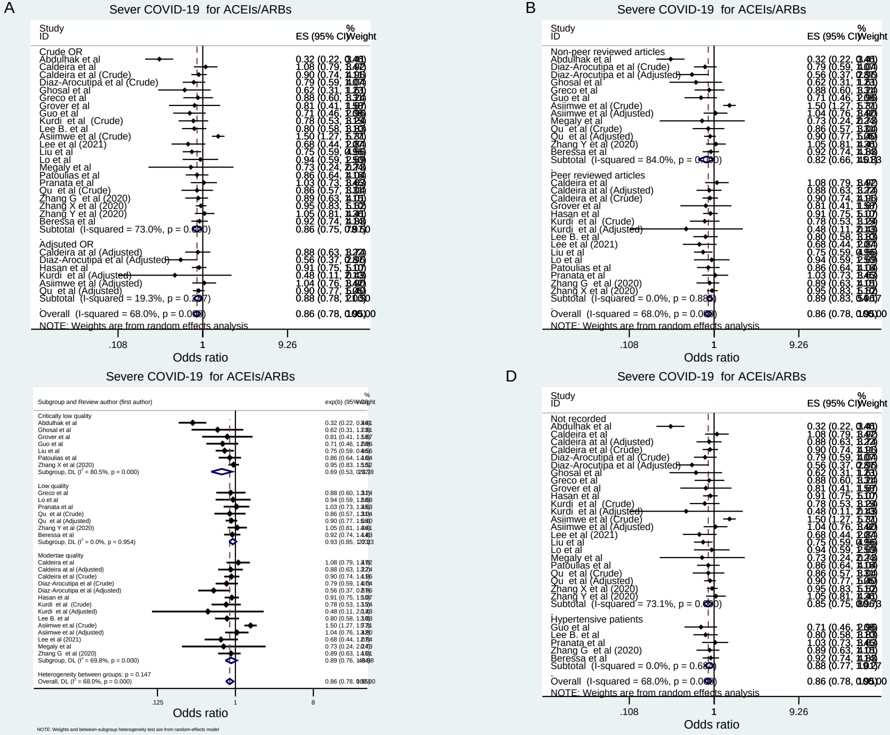
A
Sever COVID-19 for ACEIs/ARBs
Study ID
ES (95% CI)
% Weight
Crude OR
Abdulhak et al
Caldeira et al
Caldeira et al (Crude)
Diaz-Arocutipa et al (Crude)
Ghosal et al
Greco et al
Grover et al
Guo et al
Kurdi et al (Crude)
Lee B. et al
Asiimwe et al (Crude)
Lee et al (2021)
Liu et al
Lo et al
Megaly et al
Patoulias et al
Pranata et al
Qu et al (Crude)
Zhang G et al (2020)
Zhang X et al (2020)
Zhang Y et al (2020)
Beressa et al
Subtotal (I-squared = 73.0%, p = 0.000)
Adjsuted OR
Caldeira at al (Adjusted)
Diaz-Arocutipa et al (Adjusted)
Hasan et al
Kurdi et al (Adjusted)
Asiimwe et al (Adjusted)
Qu et al (Adjusted)
Subtotal (I-squared = 19.3%, p = 0.287)
Overall (I-squared = 68.0%, p = 0.000)
NOTE: Weights are from random effects analysis
.108
1
9.26
Odds ratio
B
Severe COVID-19 for ACEIs/ARBs
Study ID
ES (95% CI)
% Weight
Non-peer reviewed articles
Abdulhak et al
Diaz-Arocutipa et al (Crude)
Diaz-Arocutipa et al (Adjusted)
Ghosal et al
Greco et al
Guo et al
Asiimwe et al (Crude)
Asiimwe et al (Adjusted)
Megaly et al
Qu et al (Crude)
Qu et al (Adjusted)
Zhang Y et al (2020)
Beressa et al
Subtotal (I-squared = 84.0%, p = 0.000)
Peer reviewed articles
Caldeira et al
Caldeira at al (Adjusted)
Caldeira et al (Crude)
Grover et al
Hasan et al
Kurdi et al (Crude)
Kurdi et al (Adjusted)
Lee B. et al
Lee et al (2021)
Liu et al
Lo et al
Patoulias et al
Pranata et al
Zhang G et al (2020)
Zhang X et al (2020)
Subtotal (I-squared = 0.0%, p = 0.885)
Overall (I-squared = 68.0%, p = 0.000)
NOTE: Weights are from random effects analysis
.108
1
9.26
Odds ratio
C
Severe COVID-19 for ACEIs/ARBs
Subgroup and Review author (first author)
exp(b) (95% CI)
% Weight
Critically low quality
Abdulhak et al
Ghosal et al
Grover et al
Guo et al
Liu et al
Patoulias et al
Zhang X et al (2020)
Subgroup, DL (I² = 80.5%, p = 0.000)
Low quality
Greco et al
Lo et al
Pranata et al
Qu et al (Crude)
Qu et al (Adjusted)
Zhang Y et al (2020)
Beressa et al
Subgroup, DL (I² = 0.0%, p = 0.954)
Modertae quality
Caldeira et al
Caldeira at al (Adjusted)
Caldeira et al (Crude)
Diaz-Arocutipa et al (Crude)
Diaz-Arocutipa et al (Adjusted)
Hasan et al
Kurdi et al (Crude)
Kurdi et al (Adjusted)
Lee B. et al
Asiimwe et al (Crude)
Asiimwe et al (Adjusted)
Lee et al (2021)
Megaly et al
Zhang G et al (2020)
Subgroup, DL (I² = 69.8%, p = 0.000)
Heterogeneity between groups: p = 0.147
Overall, DL (I² = 68.0%, p = 0.000)
.125
1
8
Odds ratio
NOTE: Weights and between-subgroup heterogeneity test are from random-effects model
D
Severe COVID-19 for ACEIs/ARBs
Study ID
ES (95% CI)
% Weight
Not recorded
Abdulhak et al
Caldeira et al
Caldeira at al (Adjusted)
Caldeira et al (Crude)
Diaz-Arocutipa et al (Crude)
Diaz-Arocutipa et al (Adjusted)
Ghosal et al
Greco et al
Grover et al
Hasan et al
Kurdi et al (Crude)
Kurdi et al (Adjusted)
Asiimwe et al (Crude)
Asiimwe et al (Adjusted)
Lee et al (2021)
Liu et al
Lo et al
Megaly et al
Patoulias et al
Qu et al (Crude)
Qu et al (Adjusted)
Zhang X et al (2020)
Zhang Y et al (2020)
Subtotal (I-squared = 73.1%, p = 0.000)
Hypertensive patients
Guo et al
Lee B. et al
Pranata et al
Zhang G et al (2020)
Beressa et al
Subtotal (I-squared = 0.0%, p = 0.682)
Overall (I-squared = 68.0%, p = 0.000)
NOTE: Weights are from random effects analysis
.108
1
9.26
Odds ratio
