# Supplementary material for: An umbrella review and meta‐analysis of renin–angiotensin system drugs use and COVID‐19 outcomes
Source: Eur J Clin Invest. 2022 Oct 19;53(2):e13888. doi: 10.1111/eci.13888 (PMC9874890; doi:10.1111/eci.13888)
Supplement: Supplementary file 13 — Supplementary file S8A [file ECI-53-0-s002.pdf]

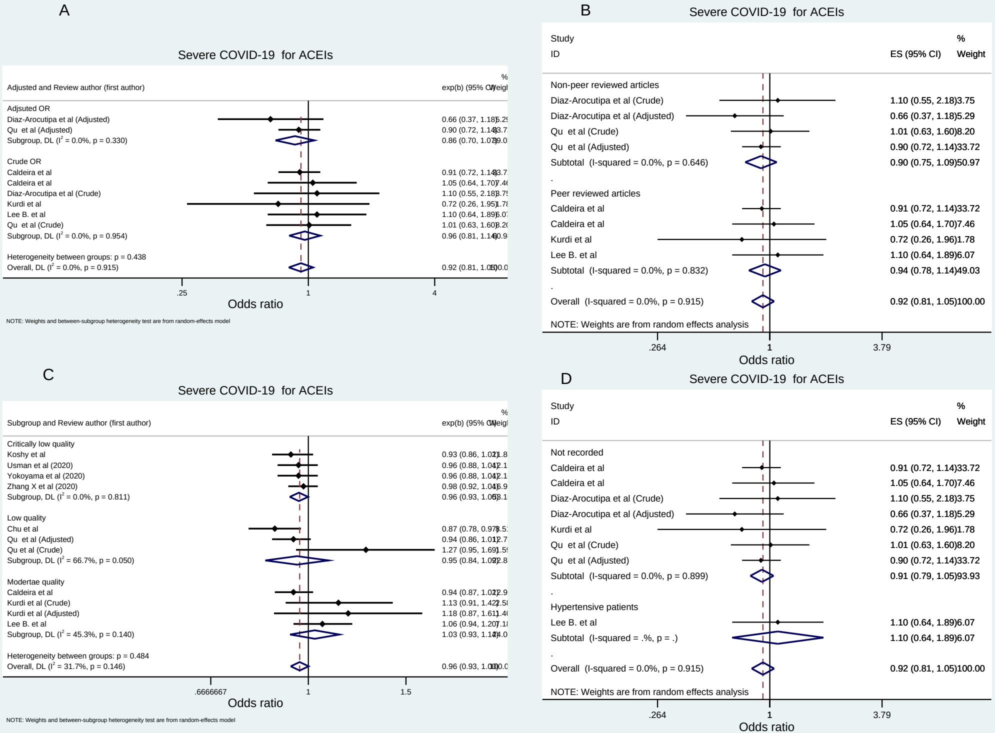
A
Severe COVID-19 for ACEIs
Adjusted and Review author (first author)
exp(b) (95% CI)
%
Weight
Adjsuted OR
Diaz-Arocutipa et al (Adjusted)
Qu et al (Adjusted)
Subgroup, DL (I^2 = 0.0%, p = 0.330)
Crude OR
Caldeira et al
Caldeira et al
Diaz-Arocutipa et al (Crude)
Kurdi et al
Lee B. et al
Qu et al (Crude)
Subgroup, DL (I^2 = 0.0%, p = 0.954)
Heterogeneity between groups: p = 0.438
Overall, DL (I^2 = 0.0%, p = 0.915)
.25
1
4
Odds ratio
NOTE: Weights and between-subgroup heterogeneity test are from random-effects model
B
Severe COVID-19 for ACEIs
Study
ID
ES (95% CI)
%
Weight
Non-peer reviewed articles
Diaz-Arocutipa et al (Crude) 1.10 (0.55, 2.18)3.75
Diaz-Arocutipa et al (Adjusted) 0.66 (0.37, 1.18)5.29
Qu et al (Crude) 1.01 (0.63, 1.60)8.20
Qu et al (Adjusted) 0.90 (0.72, 1.14)33.72
Subtotal (I-squared = 0.0%, p = 0.646) 0.90 (0.75, 1.09)50.97
Peer reviewed articles
Caldeira et al 0.91 (0.72, 1.14)33.72
Caldeira et al 1.05 (0.64, 1.70)7.46
Kurdi et al 0.72 (0.26, 1.96)1.78
Lee B. et al 1.10 (0.64, 1.89)6.07
Subtotal (I-squared = 0.0%, p = 0.832) 0.94 (0.78, 1.14)49.03
Overall (I-squared = 0.0%, p = 0.915) 0.92 (0.81, 1.05)100.00
NOTE: Weights are from random effects analysis
.264
1
3.79
Odds ratio
C
Severe COVID-19 for ACEIs
Subgroup and Review author (first author)
exp(b) (95% CI)
%
Weight
Critically low quality
Koshy et al
Usman et al (2020)
Yokoyama et al (2020)
Zhang X et al (2020)
Subgroup, DL (I^2 = 0.0%, p = 0.811)
Low quality
Chu et al
Qu et al (Adjusted)
Qu et al (Crude)
Subgroup, DL (I^2 = 66.7%, p = 0.050)
Modertae quality
Caldeira et al
Kurdi et al (Crude)
Kurdi et al (Adjusted)
Lee B. et al
Subgroup, DL (I^2 = 45.3%, p = 0.140)
Heterogeneity between groups: p = 0.484
Overall, DL (I^2 = 31.7%, p = 0.146)
.6666667
1
1.5
Odds ratio
NOTE: Weights and between-subgroup heterogeneity test are from random-effects model
D
Severe COVID-19 for ACEIs
Study
ID
ES (95% CI)
%
Weight
Not recorded
Caldeira et al 0.91 (0.72, 1.14)33.72
Caldeira et al 1.05 (0.64, 1.70)7.46
Diaz-Arocutipa et al (Crude) 1.10 (0.55, 2.18)3.75
Diaz-Arocutipa et al (Adjusted) 0.66 (0.37, 1.18)5.29
Kurdi et al 0.72 (0.26, 1.96)1.78
Qu et al (Crude) 1.01 (0.63, 1.60)8.20
Qu et al (Adjusted) 0.90 (0.72, 1.14)33.72
Subtotal (I-squared = 0.0%, p = 0.899) 0.91 (0.79, 1.05)93.93
Hypertensive patients
Lee B. et al 1.10 (0.64, 1.89)6.07
Subtotal (I-squared = .%, p = .) 1.10 (0.64, 1.89)6.07
Overall (I-squared = 0.0%, p = 0.915) 0.92 (0.81, 1.05)100.00
NOTE: Weights are from random effects analysis
.264
1
3.79
Odds ratio
