# Supplementary material for: An umbrella review and meta‐analysis of renin–angiotensin system drugs use and COVID‐19 outcomes
Source: Eur J Clin Invest. 2022 Oct 19;53(2):e13888. doi: 10.1111/eci.13888 (PMC9874890; doi:10.1111/eci.13888)
Supplement: Supplementary file 17 — Supplementary file S9B [file ECI-53-0-s013.pdf]

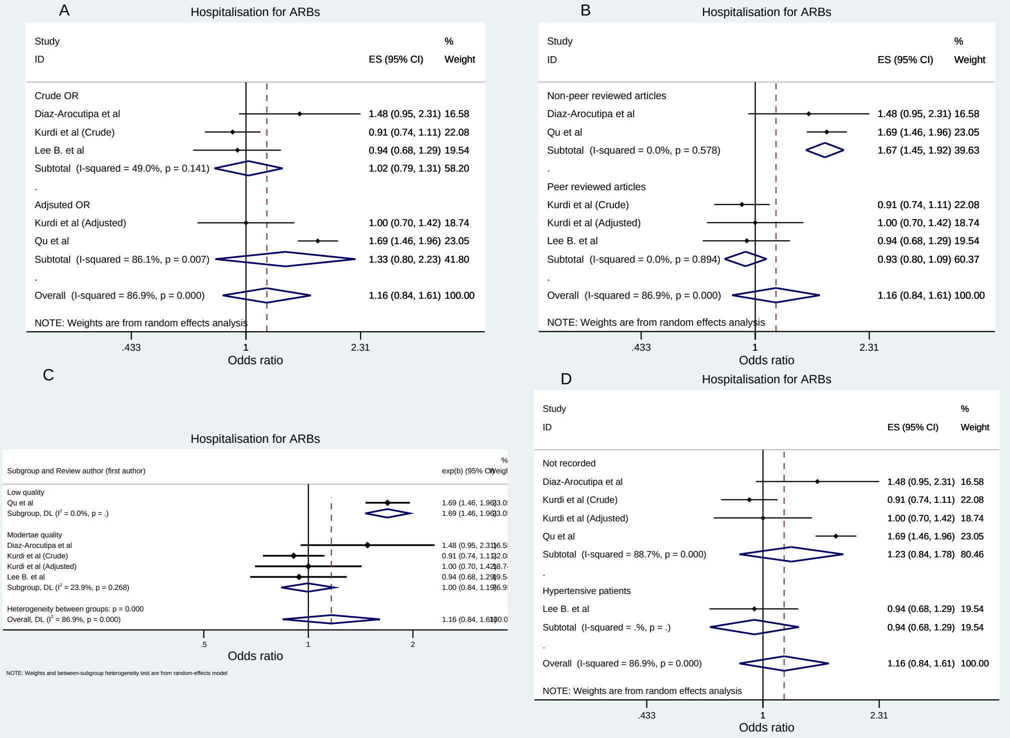

A
Hospitalisation for ARBs
Study ID
ES (95% CI)
% Weight
Crude OR
Diaz-Arocutipa et al 1.48 (0.95, 2.31) 16.58
Kurdi et al (Crude) 0.91 (0.74, 1.11) 22.08
Lee B. et al 0.94 (0.68, 1.29) 19.54
Subtotal (I-squared = 49.0%, p = 0.141) 1.02 (0.79, 1.31) 58.20
Adjsuted OR
Kurdi et al (Adjusted) 1.00 (0.70, 1.42) 18.74
Qu et al 1.69 (1.46, 1.96) 23.05
Subtotal (I-squared = 86.1%, p = 0.007) 1.33 (0.80, 2.23) 41.80
Overall (I-squared = 86.9%, p = 0.000) 1.16 (0.84, 1.61) 100.00
NOTE: Weights are from random effects analysis
.433
1
2.31
Odds ratio
B
Hospitalisation for ARBs
Study ID
ES (95% CI)
% Weight
Non-peer reviewed articles
Diaz-Arocutipa et al 1.48 (0.95, 2.31) 16.58
Qu et al 1.69 (1.46, 1.96) 23.05
Subtotal (I-squared = 0.0%, p = 0.578) 1.67 (1.45, 1.92) 39.63
Peer reviewed articles
Kurdi et al (Crude) 0.91 (0.74, 1.11) 22.08
Kurdi et al (Adjusted) 1.00 (0.70, 1.42) 18.74
Lee B. et al 0.94 (0.68, 1.29) 19.54
Subtotal (I-squared = 0.0%, p = 0.894) 0.93 (0.80, 1.09) 60.37
Overall (I-squared = 86.9%, p = 0.000) 1.16 (0.84, 1.61) 100.00
NOTE: Weights are from random effects analysis
.433
1
2.31
Odds ratio
C
Hospitalisation for ARBs
Subgroup and Review author (first author)
exp(b) (95% CI)
% Weight
Low quality
Qu et al 1.69 (1.46, 1.96) 23.05
Subgroup, DL (I² = 0.0%, p = .) 1.69 (1.46, 1.96) 23.05
Modertae quality
Diaz-Arocutipa et al 1.48 (0.95, 2.31) 16.58
Kurdi et al (Crude) 0.91 (0.74, 1.11) 22.08
Kurdi et al (Adjusted) 1.00 (0.70, 1.42) 18.74
Lee B. et al 0.94 (0.68, 1.29) 19.54
Subgroup, DL (I² = 23.9%, p = 0.268) 1.00 (0.84, 1.19) 76.95
Heterogeneity between groups: p = 0.000
Overall, DL (I² = 86.9%, p = 0.000) 1.16 (0.84, 1.61) 100.00
.5
1
2
Odds ratio
NOTE: Weights and between-subgroup heterogeneity test are from random-effects model
D
Hospitalisation for ARBs
Study ID
ES (95% CI)
% Weight
Not recorded
Diaz-Arocutipa et al 1.48 (0.95, 2.31) 16.58
Kurdi et al (Crude) 0.91 (0.74, 1.11) 22.08
Kurdi et al (Adjusted) 1.00 (0.70, 1.42) 18.74
Qu et al 1.69 (1.46, 1.96) 23.05
Subtotal (I-squared = 88.7%, p = 0.000) 1.23 (0.84, 1.78) 80.46
Hypertensive patients
Lee B. et al 0.94 (0.68, 1.29) 19.54
Subtotal (I-squared = .%, p = .) 0.94 (0.68, 1.29) 19.54
Overall (I-squared = 86.9%, p = 0.000) 1.16 (0.84, 1.61) 100.00
NOTE: Weights are from random effects analysis
.433
1
2.31
Odds ratio
