# Supplementary material for: An umbrella review and meta‐analysis of renin–angiotensin system drugs use and COVID‐19 outcomes
Source: Eur J Clin Invest. 2022 Oct 19;53(2):e13888. doi: 10.1111/eci.13888 (PMC9874890; doi:10.1111/eci.13888)
Supplement: Supplementary file 19 — Supplementary file S11 [file ECI-53-0-s012.pdf]

Supplementary file 11. Results of the influential analyses

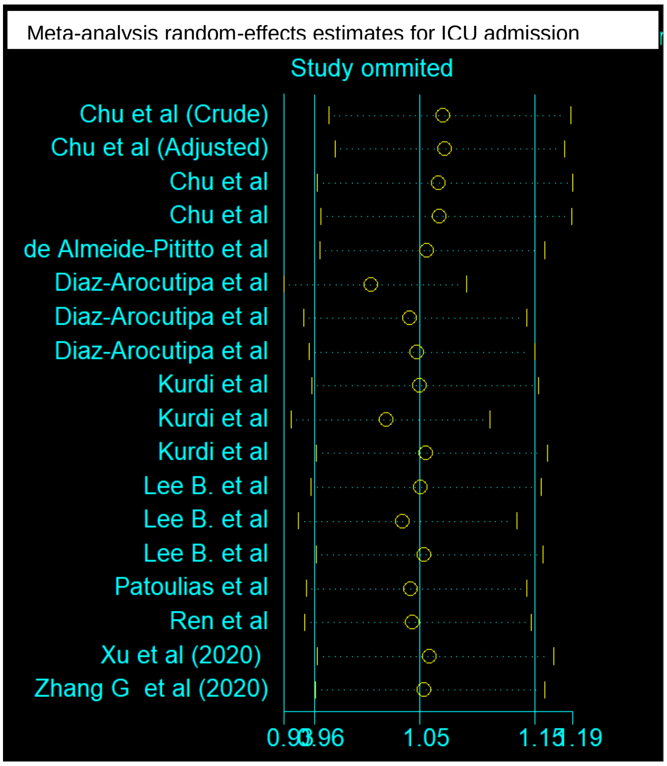

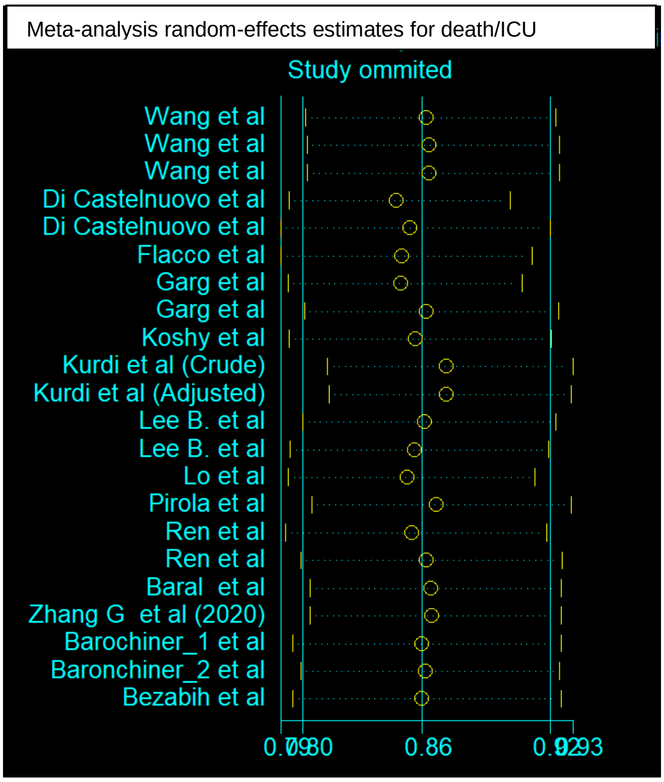

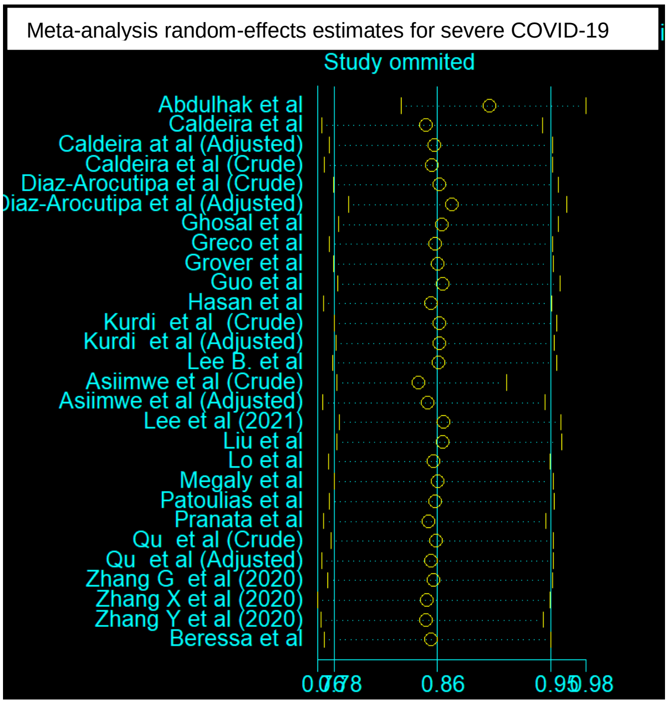
Meta-analysis random-effects estimates for severe COVID-19
Study ommited
Abdulhak et al
Caldeira et al
Caldeira at al (Adjusted)
Caldeira et al (Crude)
Diaz-Arocutipa et al (Crude)
Diaz-Arocutipa et al (Adjusted)
Ghosal et al
Greco et al
Grover et al
Guo et al
Hasan et al
Kurdi et al (Crude)
Kurdi et al (Adjusted)
Lee B. et al
Asiimwe et al (Crude)
Asiimwe et al (Adjusted)
Lee et al (2021)
Liu et al
Lo et al
Megaly et al
Patoulias et al
Pranata et al
Qu et al (Crude)
Qu et al (Adjusted)
Zhang G et al (2020)
Zhang X et al (2020)
Zhang Y et al (2020)
Beressa et al
0.86

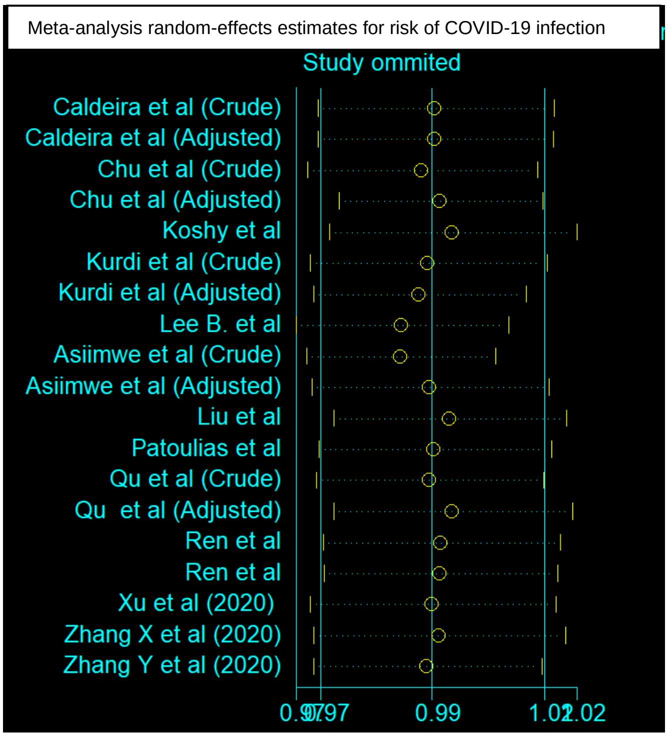
Meta-analysis random-effects estimates for risk of COVID-19 infection
Study ommited
Caldeira et al (Crude)
Caldeira et al (Adjusted)
Chu et al (Crude)
Chu et al (Adjusted)
Koshy et al
Kurdi et al (Crude)
Kurdi et al (Adjusted)
Lee B. et al
Asiimwe et al (Crude)
Asiimwe et al (Adjusted)
Liu et al
Patoulias et al
Qu et al (Crude)
Qu et al (Adjusted)
Ren et al
Ren et al
Xu et al (2020)
Zhang X et al (2020)
Zhang Y et al (2020)
0.99

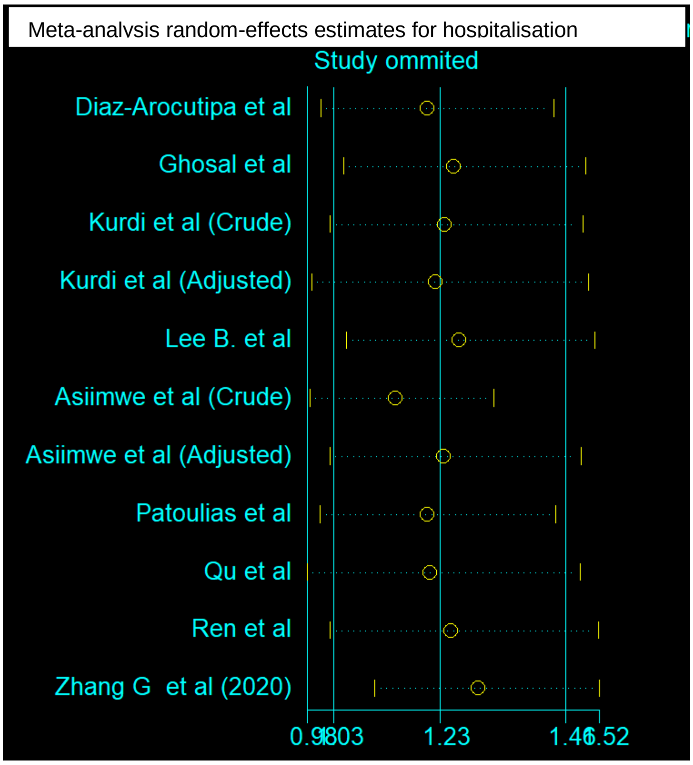
Meta-analysis random-effects estimates for hospitalisation
Study ommited
Diaz-Arocutipa et al
Ghosal et al
Kurdi et al (Crude)
Kurdi et al (Adjusted)
Lee B. et al
Asiimwe et al (Crude)
Asiimwe et al (Adjusted)
Patoulias et al
Qu et al
Ren et al
Zhang G et al (2020)
1.23
